# C-terminal sequence stability profiling in *Saccharomyces cerevisiae* reveals protective protein quality control pathways

**DOI:** 10.1016/j.jbc.2023.105166

**Published:** 2023-08-16

**Authors:** Sophia Hasenjäger, Andrea Bologna, Lars-Oliver Essen, Roberta Spadaccini, Christof Taxis

**Affiliations:** 1 Department of Biology/Genetics, Philipps-University Marburg, Marburg, Germany; 2 Department of Science and Technology, Universita’ Degli Studi Del Sannio, Benevento, Italy; 3 Department of Chemistry/Biochemistry, Philipps-University Marburg, Marburg, Germany; 4 Department of Medicine, Health and Medical University, Erfurt, Germany

**Keywords:** proteasome, proteolysis, protein quality control, C terminus, protein degradation, ubiquitin–proteasome system, optogenetics

## Abstract

Protein quality control (PQC) mechanisms are essential for degradation of misfolded or dysfunctional proteins. An essential part of protein homeostasis is recognition of defective proteins by PQC components and their elimination by the ubiquitin–proteasome system, often concentrating on protein termini as indicators of protein integrity. Changes in amino acid composition of C-terminal ends arise through protein disintegration, alternative splicing, or during the translation step of protein synthesis from premature termination or translational stop-codon read-through. We characterized reporter protein stability using light-controlled exposure of the random C-terminal peptide collection (CtPC) in budding yeast revealing stabilizing and destabilizing features of amino acids at positions −5 to −1 of the C terminus. The (de)stabilization properties of CtPC-degrons depend on amino acid identity, position, as well as composition of the C-terminal sequence and are transferable. Evolutionary pressure toward stable proteins in yeast is evidenced by amino acid residues under-represented in cytosolic and nuclear proteins at corresponding C-terminal positions, but over-represented in unstable CtPC-degrons, and vice versa. Furthermore, analysis of translational stop-codon read-through peptides suggested that such extended proteins have destabilizing C termini. PQC pathways targeting CtPC-degrons involved the ubiquitin–protein ligase Doa10 and the cullin–RING E3 ligase SCF^Das1^ (Skp1–Cullin–F-box protein). Overall, our data suggest a proteome protection mechanism that targets proteins with unnatural C termini by recognizing a surprisingly large number of C-terminal sequence variants.

Mechanisms of intracellular proteolysis and protein quality control (PQC) are essential for eukaryotic cells to maintain a functional proteome. In humans, disbalanced proteostasis is connected with the development of age-related diseases and neurodegenerative disorders (1, 2). Regulated proteolysis of supernumerous or damaged proteins as well as protein aggregates is mainly carried out by the ubiquitin–proteasome system (UPS) and autophagy–lysosome pathways (3, 4, 5, 6, 7, 8). Degradation *via* the UPS is initiated by covalent linkage of the highly conserved protein ubiquitin to a substrate protein. An enzyme cascade consisting of an ubiquitin-activating enzyme (E1), ubiquitin-conjugating enzyme (E2), and an ubiquitin-protein ligase (E3) modifies the substrate with ubiquitin creating a covalent bond between the carboxy-terminal glycine residue of ubiquitin with the side chain of a lysine (5). To initiate substrate degradation, iterative ubiquitylation takes place that results in linear or branched chains of polyubiquitin, a process that requires in some cases an additional enzyme (E4), a polyubiquitin chain conjugation factor (9). Polyubiquitylation leads to substrate recruitment by the proteasome and, eventually, unfolding and degradation of the substrate protein (3).

A crucial step in this process is the recognition of a degradation-initiating signal (degron) in the target protein, which is done by an ubiquitin–protein ligase or a chaperone acting in concert with an E3 (1, 3, 5). Degrons are defined as minimal and sufficient elements, which are responsible for recognition and degradation of a substrate. Often, a short linear stretch of amino acids is recognized and a lysine residue is used for ubiquitylation (10). Importantly, proteasomal degradation requires a stretch of about 30 unstructured amino acids to initiate degradation (11).

The protein termini have been recognized as important indicators for protein integrity; they are monitored by PQC mechanisms that recognize unusual amino acid combinations, which indicate damaged or defective proteins. A hallmark was the identification of a degron consisting of specific amino acids at the N terminus; more than 30 years of intense research has revealed a complex network of E3s involved in PQC mechanisms for the N terminus (12). Recently, it has been demonstrated that the C terminus is under surveillance as well because of the identification of a set of diverse C-degrons in mammalian cells (8, 13).

The identification and characterization of C-degrons results from work using mammalian cell culture and the eukaryotic model organism yeast. In mammalian cells, C-degrons with alanine, glutamic acid, glycine, or arginine at specific positions near the C terminus trigger degradation through recognition by E3s of the Cullin–RING E3 family (CRLs) as well as other ubiquitin–protein ligases (14, 15). CRLs are a major class of modular ligase complexes that are composed of a Cullin scaffold, a RING finger domain protein that anchors an E2, and a substrate-recognition receptor (substrate-recognition subunit [SRS]) connected to the Cullin by an adaptor protein. For example, the SCF (Skp1–Cullin–F-box protein) complexes in budding yeast consist of the adaptor protein Skp1, the Cullin scaffold Cdc53, the RING finger protein Rbx1, and modular F-box proteins as substrate receptors (16). In *Saccharomyces cerevisiae*, C-degrons have been identified by generating C-terminal extensions of reporter proteins with endogenous sequences placed out of their natural context (17, 18, 19). These degrons were mostly recognized by the ubiquitin ligases Doa10 and Ltn1 (17, 19). However, the common features of C-degrons and the deciding factors that render a protein stable or unstable are not well understood; a comprehensive stability code like the one for the N terminus is still missing for the C-terminal end of proteins. Amino acids at specific positions within a short recognition sequence have been classified as degradation signal in sequences near the carboxy terminus or comprising the last amino acids of a protein (14, 20, 21, 22, 23). Mislocalized proteins originating from the secretory pathway and mitochondria as well as products of deubiquitylating enzymes have been found to be degraded *via* C-degrons (24). It has been observed that destabilizing amino acids near the C terminus are depleted in the proteome of eukaryotic cells and that C-terminal PQC pathways shape C-terminal sequences in the human proteome (14). This raises the question, whether other features in the genome might be influenced by C-terminal PQC pathways as well.

Here, we used controlled exposure of C-terminal amino acid sequences in *S. cerevisiae* to investigate their influence on protein stability and to characterize whether specific amino acids at certain positions near the C terminus or specific amino acid sequences at the C terminus confer protein instability. Controlled exposure was achieved with an established blue light switch, a variant of the photoreceptor domain light–oxygen–voltage 2 (LOV2) of *Avena sativa* phototropin 1 (25). This domain and relatives thereof have been used previously in the context of targeted protein degradation in diverse cell lines and organisms (25, 26, 27, 28, 29, 30, 31, 32, 33). Light-induced unfolding of an α-helix at the C terminus of the LOV2 domain can be used to expose an otherwise inaccessible degron sequence (30, 34). The destabilizing influence of different C termini was characterized with a split tandem fluorescent protein reporter (STFPR) construct that contained superfolder GFP as reference reporter separated from mScarlet^I^ (red fluorescent protein [RFP]) as stability reporter by the viral P2A linker sequence, which induces separation of the two fluorescent proteins during translation. This facilitated fluorescence-based normalization of RFP amounts present in darkness compared with blue light (Fig. 1*A*). Similar STFPRs have been used previously to characterize protein degradation efficiency (25, 29, 35). The photoreceptor domain of psd3 (iLID variant of LOV2 with deletion of alanine 416 (25) was placed at the C terminus of mScarlet^I^ followed by 16 randomly chosen amino acids, which were supposed not to adopt a stable secondary fold. Efficient degradation of a protein requires roughly 30 amino acids that are unstructured to initiate degradation in the proteasome (11). Blue light–induced unfolding of the Jα helix in the LOV2 domain complements the 16 amino acids to form a potentially functional degron. The last six amino acids of this construct were arbitrarily chosen as AEAKEL. The combination of blue light–induced unmasking coupled with a random artificial C-terminal peptide collection (CtPC) was used to characterize the influence of the composition of C termini on protein stability in *S. cerevisiae*. We identified degron sequences of strong, intermediate, and weak activity as well as stabilizing sequences. Moreover, we characterized degradation pathways for these degrons and identified the SCF^Das1^ complex as a mediator of C-terminal PQC. Our findings imply that the composition of the C-terminal end of proteins is shaped toward higher protein stability and that erroneous translational read-through results in proteins with destabilizing extensions.

**Figure 1:**
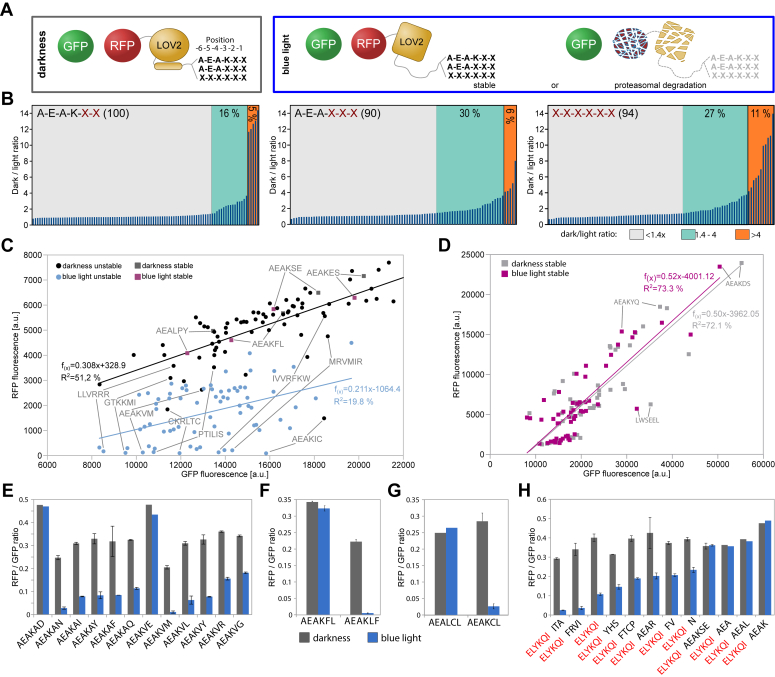
**Influence of light-exposed short random C-terminal peptides on protein stability measured with a split tandem fluorescent protein reporter.***A*, schematic of the LOV2-based system to characterize the influence of different C termini on the stability of the fluorescence reporter mScarlett^I^ (RFP). Blue light–induced unfolding of the Jɑ-helix of the LOV2 domain unmasks the C terminus and might be recognized by protein quality control. The *sf*GFP was used as a protein biosynthesis reference. The last two, three, or six amino acids of the C terminus were randomly exchanged within the GFP-P2A-RFP-LOV2 construct to generate CtPC variants. *B*, distribution of switching ratios between light conditions of randomly picked CtPC variants. Cells carrying the construct *sf*GFP-P2A-RFP-LOV2 with randomly generated C termini were randomly selected. The RFP–GFP ratio of the selected clones was determined after 5 h in darkness and blue light. The dark–light switching ratios are shown for randomly selected clones with exchanges of two, three, and six amino acids. Variants were ordered by their switching ratios. Numbers in *brackets* indicate the number of tested clones. *C* and *D*, plot of fluorescence intensity of GFP and RFP of CtPC-degrons (*C*) and stable CtPC variants (*D*). Wildtype (ESM356-1) cells containing CtPC plasmids were incubated in darkness or blue light for 5 h prior to the measurements. Selected variants are highlighted. Linear fits for both conditions are shown together with the value for *R*-squared that indicates how well the linear regression model fits the data. *E*–*H*, selected examples of CtPC variants. RFP–GFP ratios are shown in the graphs. The error bars show the standard deviation (n = 3). CtPC, C-terminal peptide collection; LOV2, light–oxygen–voltage 2; RFP, red fluorescent protein; SfGFP, superfolder GFP.

## Results

### Rare amino acids at the C terminus induce proteolysis

As starting point, we choose to analyze *in silico* the amino acid distribution at C termini of budding yeast proteins to identify amino acids that are rarely present within the last six residues. This revealed that in absolute numbers, tryptophan and cysteine are the most uncommon amino acids at C termini (Fig. S1*A*). We tested our hypothesis that rare amino acids at the C terminus trigger PQC and made single replacements of the last four positions in the STFPR. The stability of the resulting proteins was assessed in yeast cells that were kept in darkness or exposed to blue light in comparison to the parental construct with the amino acids AEAKEL at the C terminus (Fig. S1*B*). This revealed two variants that were destabilized by blue light, AEAKCL and AEWKEL and two additional sequences AEACEL and AEAKEC, with slightly reduced abundance in blue light. However, the dark–light ratio of the latter two variants was negligible with a ratio of 1.1 compared with 10.3 and 1.5 for the variants AEAKCL and AEWKEL, respectively. Thus, the variants AEACEL and AEAKEC were not investigated further. Fluorescence microscopy analysis confirmed uniform RFP fluorescence in darkness and a blue light–induced degradation of the reporter by the AEAKCL C-degron construct (Cdeg^AEAKCL^), whereas the sequence AEWKEL had only a minimal influence on abundance (Fig. S1*C*). Immunoblotting confirmed the results obtained by fluorescence measurements for the AEAKCL variant (Fig. S1*D*). As expected, shortening of the unstructured linker by removing the LOV2 domain led to stabilization of the STFPR (Fig. S2, *A* and *B*), most likely because of an insufficient initiation region that is required for proteasomal degradation (11).

### The C-terminal amino acid composition influences protein stability

The facile generation of proteolysis-inducing C-terminal sequences made us wonder, which fraction of C-terminal peptides is destabilizing and which PQC pathway is used for clearance. We addressed the former question by investigating the influence of different C termini on protein stability by generating a random artificial CtPC. Since rules for destabilizing or stabilizing C termini have not been defined for yeast, we created three different sets of random C-terminal sequences that differed only in the number of randomly exchanged positions; the last six, the last three, or the last two positions were randomized (Fig. 1*A*). The stability of randomly selected clones of the three groups was measured to estimate the number of destabilizing variants in the CtPC (Fig. 1*B*). Based on the ratio of reporter abundance under blue light and darkness, clones were divided into stable and unstable variants; a dark–light ratio of 1.4 or more was classified as destabilizing variant. Roughly 20 to 40% of all possible combinations resulted in destabilization under blue light, of which about 5 to 10% had a strong destabilizing influence. This implies that the number of potentially destabilizing C-terminal sequences is surprisingly high.

For identification of destabilizing and stabilizing C-terminal sequences, we performed a preselection step quantifying the fluorescence intensity of several thousand yeast colonies grown on solid medium to enrich the number of destabilizing sequences before performing a measurement by flow cytometry (Fig. S3*A*). The plate assay resulted in roughly 425 destabilizing and stable clones that were characterized by fluorescence measurement; a dark–light switching ratio of 1.4 and above was used to define destabilizing CtPC variants. Selected variants were characterized by sequencing resulting in 72 unique destabilizing and 62 unique stable variants (Figs. 1, *C* and *D*, and S3, *B* and *C* and Table S3). The variant AEAKCL was found among the analyzed clones, and four variants were found more than once. As expected, most of the 425 clones were in the stable category and not sequenced.

A plot of GFP *versus* RFP fluorescence showed that blue light led to a moderate reduction in GFP fluorescence, regardless of the stability of the variants (Fig. 1, *C* and *D*). Destabilizing variants in darkness-adapted cells showed a roughly linear relation between RFP and GFP, whereas such a linear relation was lost upon blue light exposure because of large changes of the RFP fluorescence (Fig. 1*C*). This indicates that the destabilizing effect of CtPC-degrons depends for most variants on exposure of the C-terminal sequences and that the C-terminal sequences exert different degron strengths. Two exceptions among the CtPC-degrons, namely CKRLTC and AEAKIC, showed reduced abundance already in darkness, yet even stronger reduction upon blue light illumination and a high dark–light switching ratio (Fig. 1*C*). Stable variants showed no or only moderate RFP reduction upon blue light illumination with a dark–light switching ratio below 1.4 and a relatively linear relation between RFP and GFP fluorescence at both conditions (Fig. 1*D*).

Next, we analyzed the sequences of stable and destabilizing CtPC variants in more detail. Analysis of reporter stability in relation to the C-terminal sequences showed that a single amino acid exchange within the C-terminal sequence could result in large changes in protein stability. In the context of two CtPC variant groups (AEAK**VX** and AEAK**AX**), amino acids in the last position that are either hydrophobic, positively charged, or polar were destabilizing to a varying degree, whereas negatively charged residues at the last position (AEAK**VE** and AEAK**AD**) were stabilizing (Fig. 1*E*). Positional effects were observed as well; swapping of two amino acids at position -2 and -1 resulted in strong destabilizing activity in case of the sequence pair AEAKFL/AEAKLF (Fig. 1*F*). Furthermore, a switch at the third position between a hydrophobic and a polar amino acid led to destabilization in case of the sequence pair AEA**L**CL/AEA**K**CL (Fig. 1*G*), whereas exchanging the cysteine to phenylalanine (AEAKFL) lead to stabilization.

Moreover, the CtPC variants provide evidence that destabilizing C-degron sequences require positioning at the C terminus. Shifting a moderately destabilizing C-terminal sequence (ELYKQI) by few amino acids resulted in changed behavior. We observed complete stabilization (ELYKQI-AEA and ELYKQI-AEAL/K), partial stabilization (ELYKQI-AEAR, ELYKQI-FV, and ELYKQI-N), unchanged behavior (ELYKQI-YHS), or stronger destabilization (ELYKQI-ITA and ELYKQI-FRVI) (Fig. 1*H*). Overall, our analysis showed that single amino acid switches changed the stability of the CtPC construct at positions −1, −2, −3, and −4 (Figs. 1, *C* and *E*–*H* and S3, *B* and *C*). Namely, these are the sequence pairs AEAKVE (stable) *versus* AEAKVR (destabilized); AEAKEL (stable) *versus* AEAKCL (destabilized); AEALCL (stable) *versus* AEAKCL (destabilized); AEAKEL (stable) *versus* AEWKEL (destabilized).

Further characterization aimed at half-lives of CtPC-degrons and estimating differences in hydrophobicity between stable and destabilized variants. For the latter, the relation between dark–light switching ratio and hydrophobicity–hydrophilicity within the last six amino acids was analyzed. Among the stable CtPCs, we found 75% hydrophilic and 25% hydrophobic variants, whereas the distribution among the destabilized CtPCs was 43.2% hydrophilic, 0.01% neutral, and 56.7% hydrophobic variants. This shows an increase of hydrophobic sequences among the destabilized CtPCs compared with stabilized CtPCs and suggests that hydrophobic C termini are in general more destabilizing compared with a hydrophilic amino acid composition (Fig. S3*D*). Quantification of the stability of 20 randomly chosen CtPC-degrons resulted in a wide variety of half-lives from 7 to 80 min. Most half-lives fell between 7 and 52 min (Fig. S4). Overall, our biased and unbiased investigation resulted in a surprisingly large number of destabilizing C-terminal sequences that result in a large spread in protein half-lives. Rare and hydrophobic amino acids near the C terminus have been identified as constituents of CtPC-degrons.

### CtPC-degrons are transferable

**Figure 2:**
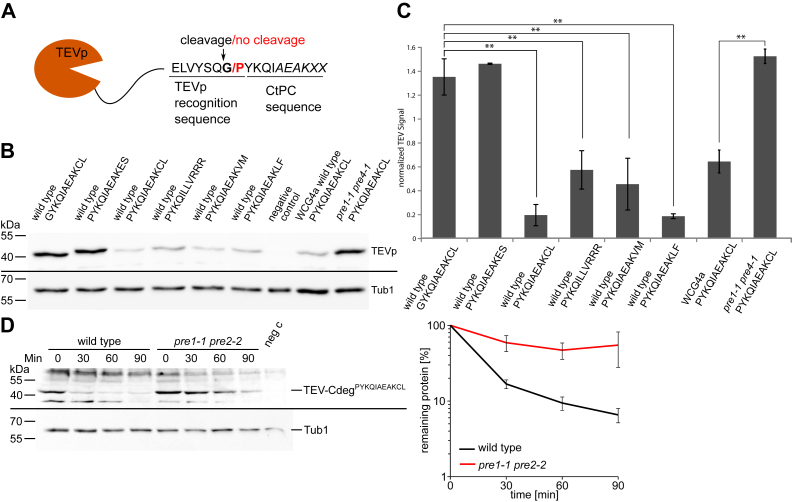
**CtPC-degrons are transferable.***A*, fusion of the last 10 amino acids of different CtPC variants to the C terminus of the TEV protease. The linker contains a TEV cleavage site (ELVYSQ-G) or a mutated sequence (ELVYSQ-P). Depending on the composition of the recognition motif, the last 10 amino acids are cleaved away (sequence with G) or not (sequence with P). *B*, immunodetection of TEV-ELVYSQ-G/P-CtPC fusion proteins in wildtype strains ESM356-1, WCG4a, and in the strain WCG4a/11/22 (*pre1-1 pre2-2*). *C*, quantification of the respective TEVp signal detected by αTEV antibody. Significance was determined *via* Student’s *t* tests (∗∗*p* < 0.01). The error bars indicate the SEM (n = 3). *D*, the translational inhibitor cycloheximide was used to stop protein synthesis in a wildtype strain (WCG4a) and a proteasomal mutant strain (*pre1-1 pre2-2*). The degradation of TEVp-PYQIAEAKCL was followed in both strains by Western blotting. The graph shows the quantification of four independent measurements of TEVp abundance normalized to Tub1p levels (n = 4; error bars = SEM). CtPC, C-terminal peptide collection; TEVp, tobacco etch virus protease.

One concern might be that the exposure of the C terminus with an LOV domain and the specific linker might create a bias for the selection process. Thus, the impact of the very last C-terminal amino acids on protein stability was investigated by using a different reporter and linker sequence. To do so, the last 10 amino acids of selected CtPCs were attached to the C terminus of tobacco etch virus protease (TEVp). The TEVp recognizes the canonical sequence ENLYFQ-G, and processing by the protease occurs then between glutamine and glycine. Exchange of glycine with proline in the recognition sequence abolishes proteolysis by TEVp (36, 37). The last 10 amino acids of selected CtPCs were fused to the C terminus of TEVp that contains a noncanonical natural recognition site (ELVYSQ-G) that is present in the viral polyprotein in an unstructured linker region downstream of the protease (38, 39). In this setting, a degron attached to a proline-containing sequence (ELVYSQ-P) stays attached, whereas the degron is cleaved away if a glycine-containing recognition sequence is used (ELVYSQ-G). Thus, these fusion proteins either expose constitutively the CtPC sequence or remove it because of TEVp activity (Fig. 2*A*). We observed the same stability properties of the C-terminal ends in the TEVp system as with the STFPR (Fig. 2, *B* and *C*). CtPC-degrons led to a destabilization of the TEV^ELVYSQP^-CtPC, whereas a stable CtPC sequence (PYKQIAEAKES) resulted in a stable TEVp-CtPC construct. If a CtPC-degron (GYKQIAEAKCL) was removed by TEVp, the protease remained stable. Moreover, we investigated whether proteolysis of destabilized variants is carried out by the proteasome using a *pre1-1 pre2-2* mutant strain with reduced proteolytic capacity of the proteasome (40). We used the TEVp construct PYKQIAEAKCL and a yeast strain with reduced proteasomal function (Fig. 2, *B* and *C*). Quantification of the immunoblot showed that reduced proteasomal function led to stabilization of the TEV^ELVYSQP^–CtPC^AEAKCL^ fusion construct (Fig. 2*C*). Moreover, we measured the degradation rate of TEV^ELVYSQP^–CtPC^AEAKCL^ in the *pre1-1 pre2-2* strain with reduced proteasomal activity compared with the corresponding wildtype strain with a cycloheximide chase. This demonstrated that the degradation of the tester protein is severely inhibited in the proteasomal mutant strain (Fig. 2*D*). Altogether, these results demonstrate that the decisive features of CtPC-degrons lie within the last 10 amino acids and are transferable.

### C-terminal composition of stable and unstable CtPC variants

**Figure 3:**
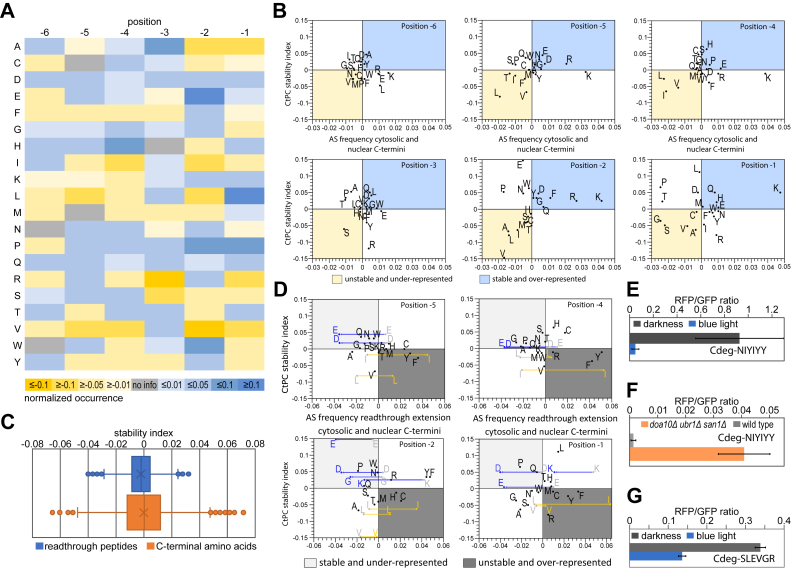
**The C-terminal peptide collection (CtPC)-stability index correlated with amino acid frequencies at the C terminus of cytosolic and nuclear proteins.***A*, the abundance of amino acids in CtPC-degron variants normalized to the occurrence in stable variants. Values above zero (shades of *blue*) indicate higher occurrence in stable CtPC variants; values below zero (shades of *yellow*) indicate increased appearance in CtPC-degrons. *B*, correlation of the CtPC-stability indices (shown in *A*) with their abundance at C-terminal positions of proteins localized in the cytosol and nucleus. The frequency of amino acids at the last six C-terminal residues was normalized to their general occurrence in cytosol and nucleus at the respective positions. The correlation of the occurrence in the yeast proteins with the CtPC stability indices indicates correlations between an over-representation or under-representation in the proteome and the frequency of occurrence in CtPC-degrons or stable variants, respectively. *C*, distribution of stability indices of first frame read-through extensions in the yeast proteome compared with the stability indices of the C termini of endogenous proteins (last six amino acids). The mean value is indicated by a *cross*, the median by a *line* within the boxplot. The *whiskers* indicate the standard deviation. *D*, correlation of CtPC-stability indices with C-terminal protein extensions because of translational stop-codon read-through. The occurrence of amino acids at the specific positions in aberrant cytosolic and nuclear protein ends was normalized by their general occurrence. The natural C-terminal frequency of selected amino acids is shown in *gray*, and the corresponding frequency of amino acids in potential read-through extensions is shown in *blue* (stability indices above zero) or *yellow* (stability indices below zero). *E*, measurement of fluorescence intensity ratio RFP/GFP of the RFP-LOV2-Cdeg^NIYIYY^ (last six amino acids of the phosphodiesterase Pde2 stop-codon read-through extension) measured in ESM356-1 cells kept in darkness or exposed to blue light. The error bars indicate standard deviation (n = 8). *F*, fluorescence intensity ratio RFP/GFP of the fusion protein RFP-LOV2-Cdeg^NIYIYY^ in BY4741 (wildtype) and YSH31 (doa10*Δ san1Δ ubr1Δ*) after 5 h blue light incubation. The error bars indicate the standard deviation (n = 3). *G*, measurement of the fluorescence intensity ratio RFP/GFP of the RFP–LOV2–CtPC^SLEVGR^ (the last six amino acids of the N-terminal fragment of the protein Scc1p after Esp1 cleavage) in dependence of the light conditions. Error bars indicate the standard deviation (n = 3). LOV2, light–oxygen–voltage 2; RFP, red fluorescent protein.

Characterization of CtPC variants allowed categorization of sequences into stabilizing or destabilizing and to relate CtPC stability to amino acid identity at the C terminus. For the latter, we generated a CtPC stability index for C-terminal amino acids at specific positions by calculating the frequency of amino acid occurrence within the last six positions in destabilized CtPC variants normalized to their abundance in stable variants (Fig. 3*A*). The composition of stable CtPC-peptides (AEAK-XX: 27%, AEA-XXX: 24.3%, and XXXXXX: 48.7%) and CtPC-degrons (AEAK-XX: 30.3%, AEA-XXX: 24.4%, and XXXXXX: 45.3%) was relatively similar in both groups, avoiding a possible bias in amino acid distribution because of the use of a C-terminal starting sequence (AEAKEL with replacement of the last two, three, or all six positions). In destabilized variants, amino acids with higher frequency were found at position −1 to be alanine, arginine, and valine, at position −2 alanine, isoleucine, leucine, and valine, at position −3 arginine and serine, at position −4 isoleucine and valine, and at position −5 leucine and valine. In stable variants, amino acids with higher frequency were found at position −1 to be leucine and proline, at position −2 glutamate, proline, and tryptophan, at position −3 alanine, and at position −4 histidine (Fig. 3*A*).

A comparative analysis of the general amino acid properties demonstrated that in destabilized CtPC variants small amino acids were preferentially found at positions −5, −2, and −1, hydrophobic or aliphatic amino acids at −2, −4, −5, and −6, and charged, positive, polar, amphipathic, and aromatic amino acids at position −3 (Fig. S5). In stable variants, negatively charged residues were more frequently found at −2 and −5, small amino acids at −3 and −4, polar and charged amino acids at −2, −4, −5, and −6, aromatic amino acids at −2, and hydrophobic and aliphatic amino acids at position −3 (Fig. S5).

Then, we looked at the normalized frequency of each amino acid and amino acid properties at specific C-terminal positions in the proteome and related it to the protein localization in the cytosol and nucleus (Fig. S6, *A* and *B*), since distinct PQC mechanisms exist in these compartments (41). This showed that lysine is over-represented at the C-terminal positions in the proteome, especially at positions −1, −2, −4, and −5. The amino acids glycine, proline, serine, and threonine are under-represented at position −1 in the proteome (Fig. S6*A*). In general, positive, charged, and polar amino acids show increased frequency at the last six positions in the proteome, whereas small amino acids are decreased (Fig. S6*B*). Correlation plots revealed a high similarity between the C-terminal residues in the two compartments with only few specific exceptions (Fig. S6*C*). In both compartments, the amino acids glycine, proline, serine, and threonine are under-represented at the position −1, like the situation in the whole proteome. At position −2, the amino acids alanine, leucine, proline, and valine are under-represented, at position −4 isoleucine, leucine, and valine, and at position −5 leucine and threonine. Most striking was the difference at position −3 for cytosolic and nuclear proteins. Here, lysine is strongly under-represented in cytosolic proteins, which contrasts the high occurrence of this amino acid at all other positions and in the nucleus at this position. In the nucleus, leucine and valine are less frequent at position −3. Other differences between the two compartments are a lowered cytosolic frequency of aspartate at position −1 and serine at position −4. In the nucleus, alanine is less frequent at position −4 than in the cytosol.

This proteome analysis of C-terminal amino acid frequencies was then used to correlate the CtPC stability indices with the amino acid distribution near the C terminus in the cytosolic and nuclear proteome (Figs. 3*B* and S7). This demonstrated that the occurrence of destabilizing amino acids at specific C-terminal positions of CtPC-degrons anticorrelates with the appearance of these amino acids at these positions within proteins localized in the yeast cytosol and nucleus (Fig. 3*B*). For example, high abundance of valine and leucine in CtPC-degrons at positions −5 and −2 anticorrelates with their presence in cytosolic and nuclear localized proteins (Fig. 3*B*). In contrast, higher occurrence of leucine at position −1 in stable CtPC variants correlates with an increased presence of leucine at position −1 compared with other C-terminal position in cytosolic and nuclear proteins. In general, hydrophobic and aliphatic amino acids are destabilizing and under-represented at positions −5 and −2, whereas charged and polar amino acids at these positions are stabilizing and over-represented (Fig. S7). In addition, the frequent occurrence of very small amino acids (Ala, Cys, Gly, and Ser) in CtPC-degrons anticorrelates with the presence in cytosolic and nuclear proteins at position −1 and, except for glycine, also at position −2 (Fig. 3*B*).

### Translational stop-codon read-through extensions generate proteins with potentially destabilizing C termini

In natural settings, the activity of inherent nonconstitutive degrons is controlled by diverse mechanisms; they might be exposed by protease cleavage, nonsplicing of a splice site, or stop-codon read-through (42, 43, 44). First, we compared the first-frame read-through extensions that may extend a protein after erroneous translation until the next stop codon is reached with the normal protein termini in the yeast proteome; for both cases, the last six amino acids were considered. This revealed that the read-through extensions had, on average, a lower stability score than the endogenous protein termini (Fig. 3*C*). Correlation of the CtPC stability indices of amino acids at specific positions with the occurrence in possible read-through extensions in cytosolic and nuclear proteins showed that destabilizing amino acids that are under-represented in the natural proteins occur more frequently in the potential read-through extension (Fig. 3*D*). The amino acids leucine and isoleucine are more abundant in potential read-through extensions at positions −5, −4, and −2 than in regular cytosolic and nuclear protein termini. In contrast, some amino acids with a stabilizing CtPC index, such as glutamate or aspartate, are less abundant at these positions in potential read-through extensions than in normal protein ends in the cytosol and nucleus. We then looked in more detail on roughly 300 highly expressed proteins, which show a prevalence for translational stop-codon read-through and contain disordered residues at the C terminus (45). Comparison of the last six amino acids of the natural protein with the extended proteins showed also a shift to more destabilizing residues (Fig. S8*A*). A plot of the CtPC stability index of the natural *versus* the extended C terminus showed that 39% of the proteins showed a shift to more stabilizing amino acids, 5% showed no or marginal difference, and 56% had a shift to more destabilizing amino acids (Fig. S8*B*). This demonstrates that a majority of proteins show a shift to more destabilizing amino acids. Thus, translational read-through of stop codons is for some proteins a constant source of erroneous proteins that might be recognized and degraded by the UPS *via* PQC components monitoring the C termini of proteins.

We tested this hypothesis using the last six amino acids of the translational read-through extension of cAMP phosphodiesterase Pde2. Pde2 is frequently produced with a C-terminal read-through extension of 20 amino acids. This extended protein is then degraded *via* the proteasome (42). To investigate the influence of a conditionally generated read-through extended C terminus, the last six amino acids of the extended phosphodiesterase Pde2 was fused to an optogenetic STFPR. Blue light–induced exposure of these amino acids destabilized the construct (Fig. 3*E*). Investigation of the degradation pathway revealed reduced degradation of the reporter in *doa10Δ ubr1Δ san1Δ* cells compared with wildtype cells under blue light (Fig. 3*F*). This finding suggests a PQC mechanism for targeted elimination of incorrectly terminated proteins.

### Generation of a potential C-degron by Esp1 cleavage of Scc1

The cohesin subunit Scc1p is known to be cleaved by the separase Esp1 (Fig. S9*A*), resulting in an exposed arginine at the N terminus that is an N-degron; this leads to rapid degradation of the C-terminal fragment of Scc1p (44). In comparison, the resulting N-terminal fragment of Scc1p is degraded at a slower pace (46, 47). The degradation sequence destabilizing the N-terminal fragment has not been identified so far. We investigated whether protease cleavage of Scc1p may generate a CtPC-like C-degron. Therefore, we tested the influence of the last six amino acids of the Esp1 cleavage site (SLEVGR; the novel C terminus after cleavage) on the stability of the STFPR (Fig. S9*B*). This revealed a blue light–dependent destabilization of the reporter construct with a switching ratio of about 2.5 (Fig. 3*G*). A cycloheximide chase analysis of the RFP-LOV2^SLEVGR^ construct showed a degradation rate of roughly 60 min in blue light–illuminated wildtype cells, which is slightly decreased in an *uba1*^ts^ strain (Fig. S9*C*). Thus, the last six residues of the N-terminal Scc1 fragment after Esp1 cleavage are sufficient to destabilize the STFPR.

### CtPC-degrons are degraded by the UPS

**Figure 4:**
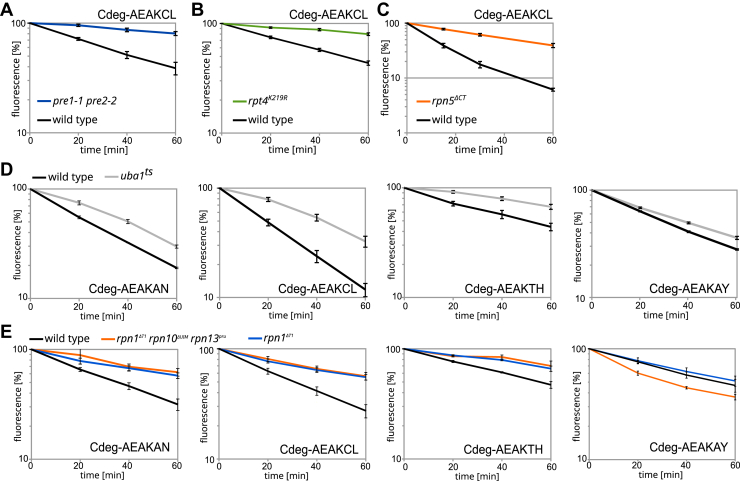
**Degradation pathways of CtPC-degrons.** Cycloheximide chases were performed in different yeast strains. Yeast cells were incubated in darkness for 5 h, protein biosynthesis was stopped by addition of cycloheximide, and yeast were incubated in blue light for 1 h. Within this period, samples were taken at the indicated time points, and the fluorescence intensity was measured by flow cytometry. Temperature-sensitive yeast strains were shifted to the restrictive temperature (37 °C) before the start of the experiment. *A*, cycloheximide chase analysis of CtPC-degron AEAKCL in WCG4a (wildtype) and WCG4a 11/22 (*pre1-1 pre2-*2) strain (n = 3); *B*, in Sub62 and DY219 (*rpt4*^*K219R*^) strains (n = 6); and *C*, in JD47-13C and AM190 (*rpn5*^*ΔCT*^) strains (n = 6). *D*, cycloheximide chase analysis of different CtPC-degrons in JD47-3C (wildtype) and JD77 (*uba1*^ts^) strains under blue light (n = 3). *E*, cycloheximide chase of CtPC-degrons in YYS40 (wildtype) and ubiquitin receptor mutant strains YDAK36 (*rpn1*^*ΔT1*^) and YDAK47 (*rpn1*^*ΔT1*^*rpn13*^*ΔUIM*^*rpn13*^*pru*^). The error bars indicate the SEM (n = 3). CtPC, C-terminal peptide collection.

Next, the degradation mechanism for destabilized CtPC variants in the STFPR was investigated in more detail. For this purpose, we used the C-degron^AEAKCL^ as a model substrate. Proteasomal degradation of RFP–LOV2–CtPC^AEAKCL^ was tested in the proteasomal mutant *pre1-1 pre2-2* that shows reduced proteolytic capacity (40). This revealed increased stability of the degron construct (Fig. 4*A*). In addition, degradation of this construct was tested in cells containing mutations in the proteasomal AAA-ATPases Rpt1–6 (48). The *rpt4* mutant showed the slowest degradation rate, most other *rpt* mutants had at least a certain impact on the CtPC^AEAKCL^ degradation rate, whereas the *rpt1* mutant showed no significant change compared with wildtype cells (Figs. 4*B* and S10). Then, we used an Rpn5 variant lacking the C terminus with weakened attachment of the proteasomal lid to the base particle (49). In the *RPN5* mutant, degradation of CtPC^AEAKCL^ is impaired (Fig. 4*C*). The lid complex of the proteasome is important for deubiquitination of substrates (50), and the interaction between lid and base is important for structural changes of the proteasome complex necessary for engagement, unfolding, and entering of substrates in the proteolytic particle (10, 51, 52). In summary, our characterization revealed that proteasomal activity and a fully assembled proteasomal complex is required for degradation of the RFP–LOV2–CtPC^AEAKCL^ construct.

To directly characterize ubiquitin dependence of destabilized CtPC variants, we used a temperature-sensitive *UBA1* yeast strain and investigated degradation of a set of 20 CtPC-degrons in this and the corresponding wildtype. This revealed a slower degradation at restrictive conditions upon blue light illumination for all tested RFP–LOV2–CtPC variants, whereas the two stable variants AEAKEL and AEAKSE did not show a decreased degradation rate in the *UBA1* mutant strain (Figs. 4*D* and S11, *A* and *B*). The CtPCs SVSFPG, AEAKIY, AEAKSH, AEAKAF, and AEAKAY showed only a weak or a moderate stabilization in the temperature-sensitive *uba1* mutant cells (Fig. S11*A*).

The necessity of ubiquitin for recruitment to the proteasome was indirectly confirmed by using yeast strains with mutations in ubiquitin-binding motifs in the proteasomal ubiquitin receptors Rpn1, Rpn10, and Rpn13 (Figs. 4*E* and S12*A*). Here, mutation within the ubiquitin-binding motif of the receptor Rpn1 resulted in the slowest degradation rate of the RFP–LOV2–CtPC^AEAKCL^, RFP–LOV2–CtPC^AEAKAN^, and RFP–LOV2–CtPC^AEAKTH^ variants (Fig. 4*E*). Thus, the receptor Rpn1 appears to be the major recruitment factor for the tested CtPC-degrons. Interestingly, Uba1 inactivation had only a weak impact on degradation for the CtPC-degron^AEAKAY^, and mutations in the proteasomal ubiquitin receptors had none (Fig. 4, *D* and *E*). We tested the degradation of CtPC^AEAKAY^ in a *PEP4 PRB1 PRC1* triple mutant strain in which the main vacuolar proteases are defective and vacuolar protein turnover is absent (53, 54). Our measurement did not indicate an influence of the vacuolar proteases on degradation of CtPC^AEAKAY^ (Fig. S12*B*).

### C-degrons mediate reporter destabilization by different E3s

Next, we investigated which ubiquitin-protein ligases are involved in degradation of destabilized CtPC variants. For this purpose, we tested the degradation of CtPC-degrons in strains lacking the E3s Doa10, San1, and Ubr1, which are key players in the cytosolic and nuclear PQC (41).

**Figure 5:**
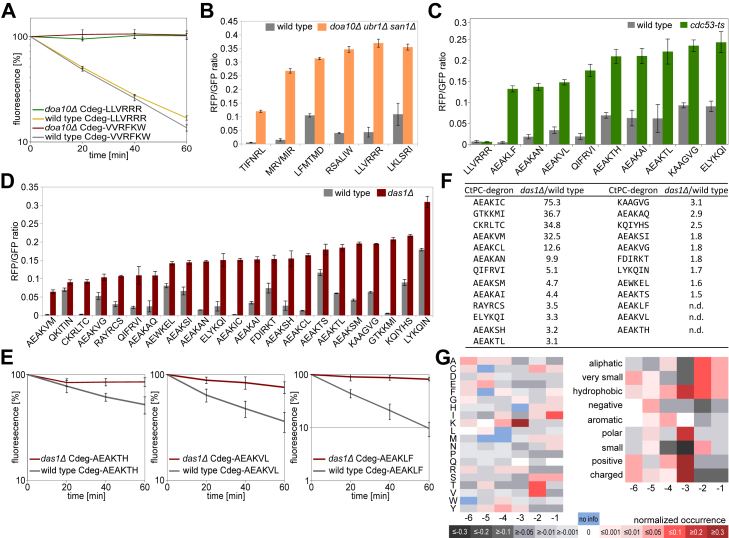
**Identification of ubiquitin–protein ligases involved in CtPC-degron degradation.***A*, cycloheximide chase of C-degrons in BY4741 (wildtype) and MHY1631 (*doa10Δ*) strain under blue light. Error bars show the standard deviation (n = 6). *B*, RFP/GFP fluorescence intensity ratios of RFP–LOV2–CtPC variants in BY4741 (wildtype) and YSH31 (*doa10Δ san1Δ ubr1Δ*) cells after 5 h blue light incubation. *C*, RFP/GFP ratios of RFP–LOV2–CtPC fusions in wildtype and TSA974 (*Cdc53*^*ts*^) cells. After 3 h of incubation, cells were shifted for 2 h to 37 °C. *D*, RFP/GFP ratios of RFP–LOV2–CtPC fusions in BY4741 (wildtype) and Y01276 (*das1Δ*). *E*, cycloheximide chases of CtPC-AEAKLF, CtPC-AEAKVL, and CtPC-AEAKTH variants in wildtype and Y01276 (*das1Δ*) cells. Error bars show the standard deviation (n = 6). *F*, overview of CtPC-degrons classified as mainly Das1 dependent based on the Y01276 (*das1Δ*) to wildtype ratio. A ratio greater than 1.5 was classified as mainly Das1-dependent degradation, one between 1.5 and 1.2 was considered a slight Das1-influence degradation, and a ratio less than 1.2 was classified as Das1-independent degradation (Table S3). *G*, appearance of amino acids at specific positions and amino acid properties in Das1-dependent C-degrons (*das1Δ*/wildtype: >1.5) normalized to Das1-independent C-degrons (*das1Δ*/wildtype: <1.2) and stable CtPC variants. Values above zero (shades of *red*) indicate increased occurrence in Das1-dependent C-degrons, and values below zero (shades of *black*) indicate increased occurrence in stable variants or Das1-independent degrons. CtPC, C-terminal peptide collection; LOV2, light–oxygen–voltage 2; n.d., not determined; RFP, red fluorescent protein.

First, the impact of a *DOA10* deletion was tested on four randomly selected CtPC-degrons by cycloheximide chases. The CtPC^AEAKLF^ and CtPC^AEAKVM^ constructs showed a slightly slowed degradation rate in *doa10Δ* cells (Fig. S13*A*), whereas the CtPC^LLVRRR^ and CtPC^VVRFKW^ variants were completely stabilized in this yeast strain under blue light (Fig. 5*A*). To determine whether the ligases San1 and Ubr1 have an influence on the mainly Doa10-independent CtPC^AEAKLF^ degradation, *san1Δ* and *ubr1Δ* strains were tested. Cycloheximide chases revealed no impact of an *SAN1* or *UBR1* deletion on the CtPC^AEAKLF^-mediated degradation (Fig. S13, *B* and *C*).

Second, CtPC variants were measured in a triple deletion strain lacking the E3s Doa10, Ubr1, and San1 by ratiometric measurements of STFPRs in blue light–illuminated cells. Of the 72 CtPC-degrons tested, five degrons as well as the control construct CtPC^LLVRRR^ showed severely decreased degradation in *doa10Δ ubr1Δ san1Δ* cells exposed to blue light (Fig. 5*B*). Thus, the ligase Doa10 was identified as the main responsible ubiquitin-protein ligase for two CtPC-degrons (LLVRRR and VVRFKW). The five CtPC-degrons, LKLSRI, LFMTMD, RSALIW, TIFNRL, and MRVMIR, require either Doa10, San1, or Ubr1 or more than one of these E3s for degradation.

Since CRL complexes in mammalian cells have been shown to be involved in the recognition and degradation of proteins with unusual C termini, we investigated the influence of the SCF complex in yeast, which is homologous to CRL1 in mammalian cells (16). The stability of CtPC-degrons not assigned to an E3 was tested in a *cdc53*^ts^ mutant strain, which is the organizing scaffold of the SCF complex in yeast (16). Under restrictive conditions, higher blue light steady-state levels were found for 9 of 38 tested CtPC-degrons in the *cdc53*^ts^ strain compared with the corresponding wildtype strain (Fig. 5*C*). SCF complexes have a set of specific SRSs that select specific target proteins. Three CtPC-degrons that displayed decreased degradation in the *cdc53*^ts^ strain were used to identify the SRS used for substrate selection (Fig. S13, *D*–*F*). This revealed increased steady-state level in a *das1Δ* strain for the selected CtPC-degrons (Fig. 5*D*). Degradation rates of three selected C-degrons (AEAKTH, AEAKVM, and AEAKLF) were severely inhibited in *das1*Δ cells (Fig. 5*E*). The stability of CtPC-degrons that were stabilized in the *cdc53*^*ts*^ strain and CtPC-degrons not assigned to another E3 was tested in a *DAS1* deletion strain. This revealed that the degradation of 25 of 75 CtPC-degrons was strongly influenced by a *DAS1* deletion, which was defined by a *das1Δ*/wildtype ratio greater than or equal to 1.5 (Fig. 5, *D* and *F*).

Next, we compared the amino acid composition between SCF^Das1^-dependent degrons with degrons recognized by other E3s and stable CtPC variants (Fig. 5*G*). Normalization of the amino acid frequency at specific positions in the SCF^Das1^-dependent CtPC-degrons with the pooled group consisting of SCF^Das1^-independent C-degrons and stable CtPCs revealed that isoleucine was often present at position −1, valine or threonine at position −2, and lysine at position −3 in SCF^Das1^-dependent CtPC-degrons. Regarding amino acid properties, very small, aliphatic, and hydrophobic amino acids preferentially occur at positions −2 and −1, whereas at position −3, preferentially, hydrophobic, polar, positive, or charged amino acids are found in SCF^Das1^-dependent substrates. Taken together, our data suggest that the last three positions in CtPC variants are the most critical for Das1-dependent degradation. This analysis matched the consensus sequence obtained by sequence alignment: t.hK.h at 80% consensus (t: turnlike amino acid; .: undefined; h: hydrophobic; and K: lysine; Fig. S14).

## Discussion

### Features of destabilizing C termini

In this study, we aimed to investigate mechanisms of cytosolic and nuclear PQC in yeast by characterizing the stability of a library of C-terminal peptides. The dynamic optogenetic approach allowed identification of amino acid residues at the C terminus that influence protein stability. In addition to strongly destabilizing sequences, this revealed weak degrons and stable sequences that are more difficult to distinguish using binary growth analysis approaches. The influence on protein stability of the last amino acids was transferable for the tested sequences. Together with an unstructured linker that contains lysine residues, 10 C-terminal amino acids were enough to comprise a fully functional degron. Even shorter C-degrons have been described (17); our characterization of stabilizing and destabilizing features of CtPCs indicated that the critical degron motifs lie within the last five to six residues. The composition of the C-terminal amino acids influences protein stability, and single amino acid exchanges or positional switch of two amino acids might result in significantly altered protein stability.

Recent studies with yeast PQC degrons have shown that in general an exposed stretch of hydrophobic amino acids without negative charges constitutes a degron (20, 55, 56) and that in mammalian cells similar rules apply (14). This is in agreement with the notion that hydrophobic amino acids are mostly embedded in the interior of globular proteins (57). We also observed that in general stable CtPC variants have more hydrophilic C termini than CtPC-degrons (Fig. S3*D*). Among the amino acids that appear with high frequency at many positions in destabilizing variants are phenylalanine, isoleucine, leucine, methionine, arginine, valine, and tyrosine. Yet, specific examples of CtPC sequences (unstable AEA**KCL** *versus* stable variants AEA**LCL**, AEA**KFL**) demonstrated that a more hydrophobic C terminus does not automatically lead to destabilization and that hydrophobic amino acids at or near the C terminus are not *per se* a signal recognized by the PQC machinery in yeast. Hydrophobic amino acids were frequently found in stable CtPC variants at position −3, whereas they were often found in destabilized variants at position −2 (Fig. S5). The data demonstrate that positioning of hydrophobic amino acids within a C-terminal sequence is important for the recognition by the PQC and that complex patterns govern recognition of destabilizing C termini in the proteome.

Negatively charged amino acids might act as stabilizing factors in protein sequences. It has been reported that the use of a negative amino acid at position −1 (exchange Q to E) within a degron sequence leads to substrate stabilization and loss of interaction with the mammalian ubiquitin–protein ligase TRIM7 (22) as well as that negatively charged amino acids are generally scarce in yeast degrons and counteract degradation signals when placed in the core of a degron (55). We observed aspartate at all six positions associated with stable variants. Yet, stabilizing effects of negatively charged amino acids were not absolute in the context of CtPC variants. Destabilization was observed in some cases despite negatively charged amino acids, regardless of whether the amino acids were located directly at position −1 (MVGFSE, LFMTMD, and TIDCAE) or near the C terminus, positions −3 (QIAEAR) or −2 (LFVAEK) (Fig. S3, *B* and *C*). Thus, the influence of negatively charged amino acids seems to be more complex and might well be sequence specific.

The generation of destabilizing C-terminal peptides was surprisingly easy, and our approach revealed that more than 30% of C-terminal peptides were weakly or strongly destabilizing. For the exchange of the last two amino acids, roughly 20 of 400 possible combinations could be strongly destabilizing and 60 sequences weakly destabilizing, whereas the majority of 320 combinations should not exert a negative on protein stability. However, a destabilizing impact in the biological context depends on several factors: a ubiquitin-dependent degron consists not only of an accessible substrate recognition motif but also of a site for ubiquitination and a suitable initiation region consisting of roughly 30 unfolded amino acids (11, 58). In yeast, about 17% of all protein sequences are predicted to be disordered, of which one-third are longer than 30 amino acids (59). In addition, the termini in eukaryotic proteins contain often exposed and disordered sequences that could act as initiation regions (60, 61, 62).

A conserved feature between human cells and yeast as well as other eukaryotes is the shaping of the proteome toward occurrence of stable sequences at the C terminus (14). The correlation/anticorrelation between amino acids within CtPC sequences and the appearance in yeast proteins indicates that certain amino acids at specific C-terminal positions or amino acid combinations within the last few residues have an impact on protein stability. The distribution of amino acid properties in CtPC and the representation of these in cytosolic and nuclear proteins correlate at positions −5, −4, −2, and −1. No clear correlation was found for positions −3 and −6; the amino acids cluster around the zero point in cytosolic and nuclear proteins at these positions (Figs. 3*B* and S7). Analysis of CtPC variants for single amino acid exchanges that destabilize a construct demonstrated that positions −4, −3, −2, and −1 were involved in defining the stability of a protein. Overall, our data indicate that the last five residues in a protein are important for protein stability *via* C-degrons. This implies that yeast proteins exposed to PQC machinery in the cytosol or the nucleus have evolved toward the occurrence of stabilizing amino acid residues near the C terminus. However, other mechanisms may also have an influence on C-terminal composition of a specific protein. For example, C-degrons can be shielded because of an interaction partner or because of an overlap with a protein–protein interaction motif (63, 64). Proteins containing a destabilizing sequence but lacking an unstructured initiation region of sufficient length remain stable (11). Thus, the composition of the C-terminal sequence of individual proteins might be under diverse constrains and may not favor in each case the generation of a stabilizing sequence.

### Degradation pathways for C-degrons

A central element in recognition of unnatural C termini are the E3s in the cytosol and the nucleus that select proteins with exposed degrons and target them for degradation. Characterization of CtPC-degron proteolysis revealed that fully functional proteasomes and ubiquitylation are required, which suggests a recognition by an E3 or a chaperone working together with an E3. Two distinct pathways involved in PQC of CtPC-degrons and both recognizing distinct C-terminal amino-acid sequences were identified: the E3s Doa10 and SCF^Das1^. The CtPCs AEAKLF and AEAKVM depend mainly on the SCF^Das1^ but are also partially dependent on Doa10. Similar observations have been made for other PQC pathways as well (41, 65, 66). This suggests that C-degrons are frequently recognized by more than one E3. The majority of C-degrons identified in yeast with cytosolic reporter variants was recognized by the ubiquitin ligases Doa10 and Ltn1 (17, 19), whereas Ubr1 and San1 were less involved in cytosolic C termini PQC (19). We observed that roughly 10% of CtPC-degrons are degraded *via* the ligases Doa10, San1, or Ubr1; two of these were targeted by Doa10. The majority of the assigned CtPC-degrons (26) was degraded *via* the SCF and Das1 as SRS. This suggests that the selection process has huge influence on the type of degrons that are identified. The chosen approach led to the identification of several weakly destabilizing sequences that are difficult to find with growth-based selection approaches.

The identification of the yeast SCF complex containing Das1 as substrate receptor as PQC pathway involved in recognition of destabilizing C-terminal sequences demonstrates that this is a conserved process in eukaryotes. Like in mammalian cells, the family of CRLs also plays a role in identification of aberrant C termini in yeast. Interestingly, Das1 does not seem to have orthologs among known human C-degron receptors.

A focus was put on the Das1 recognition sequence, as we identified 25 sequences that induce Das1-dependent degradation. The positively charged amino acid lysine was part of the fixed sequence (AEAK-XX). Analysis of all sequences that require Das1 for degradation suggested a positive charge at position -3 as well as a hydrophobic amino acid at -4 and -1 (Figs. 5*G* and S14). Further analysis should clarify the question, how strict this pattern is, as the sequences KAAGVG, LYKQIN, or KQIYHS deviate from it. Moreover, the protein Das1 was previously identified as the SRS for the C terminus of Hac1 that is formed by unspliced *HAC1* mRNA during noninducing conditions (43). The C terminus of unspliced Hac1p MTRKLQ also has a lysine at position -3 but deviates somewhat from the consensus sequence identified by our analysis.

The protein half-lives of three Das1-dependent CtPC-degrons (Fig. 5*E*) suggest different binding affinities of Das1 with C-terminal peptides depending in these cases on the composition of the last two C-terminal amino acids. Similarly, we observed residue specificity of SCF^Das1^ recognition: CtPC^AEAKLF^ and CtPC^AEAKVL^ require Das1 for degradation, whereas CtPC^AEAKFL^ was stable (Figs. 1*F* and 5E); degradation of CtPC^AEAKAI^ and CtPC^AEAKAN^ requires the SCF^Das1^ complex (Fig. 5*D*), whereas degradation of CtPC^AEAKAF^ and CtPC^AEAKAY^ was not diminished in a *das1Δ* mutant or in a *doa10Δ san1Δ ubr1Δ* triple mutant. For some CtPC-degrons like QKITIN, the *DAS1* deletion had a slight influence on the steady-state level resulting in a *das1Δ*/wildtype switching factor between 1.2 and 1.5 (Table S3). The behavior of these variants could be explained by low Das1 affinity of recognition sequence, targeting by an additional degradation pathway (*e.g.*, the E3 Ltn1), detection by more than one E3 (*e.g.*, the SCF complex, and one of the E3s Doa10, San1, or Ubr1), or ubiquitin-independent degradation by the proteasome. Careful analysis of all CtPC-degrons without clearly assigned degradation pathway is required to clarify proteolysis pathways and to identify all E3s involved in supervising C termini.

The vast majority of tested CtPC-degrons required Uba1 activity for degradation and a fully active proteasome. However, the CtPCs SVSFPG, AEAKIY, AEAKSH, AEAKAF, and AEAKAY did not show a pronounced stabilization in *uba1* mutant cells, which could indicate ubiquitin-independent proteasomal degradation. For the degron CtPC^AEAKAY^, only a weak dependency on ubiquitylation was found and no stabilization in proteasome mutants with defects in the main ubiquitin receptors. Though, an influence of vacuolar proteases on degradation of CtPC^AEAKAY^ was not detectable. One possible explanation is that the CtPC^AEAKAY^ is only partially dependent on ubiquitylation for proteolysis, like the degradation of the photosensitive degron module (31). Also, an alternative proteasomal ubiquitin receptor might be involved in degradation of the CtPC-degron^AEAKAY^. It was observed that yeast cells with mutations in the ubiquitin receptors Rpn1, Rpn10, and Rpn13 are still able to degrade selected protein substrates, which implies that additional ubiquitin receptors are existing that bind these substrates (67). A prime candidate for this additional ubiquitin receptor is the proteasomal subunit Dss1/Sem1, which contains an unstructured ubiquitin-binding domain (68). As we did not observe stabilization of the CtPC-degron^AEAKAY^ by a *DAS1* deletion or in a *DOA10 SAN1 UBR1* triple mutant, the degradation pathway for this variant is not fully clarified. Ubiquitin-independent degradation is common in mammalian cells (69). Recently, ubiquitin-independent degradation of GFP fusions of peptides derived from the C-terminal end of human proteins has been described (70). This study identified alanine at positions -1 and -2, cysteine at positions -1 and -2, as well as valine at positions -1, -2, -3, and -4 as signals for ubiquitin-independent degradation in human cells. In yeast, such a clear distinction between ubiquitin-dependent and Das1-dependent degradation does not seem to be in operation. Although the occurrence of alanine at the last three positions is reduced in Das1-dependent CtPCs, the occurrence of cysteine and valine at position -2 is increased. Overall, there are interesting similarities between C-degron pathways in human and yeast cells. On the level of the molecular organization, differences are apparent for the degradation mechanism of specific proteins and their degron sequences.

A remaining question is how many of the newly identified C-degrons need to have the degron sequence located at the very C terminus of the protein to initiate degradation and how many of these would also act as degron at other locations within a polypeptide sequence. For N-degrons, this question was solved by revealing the recognition mechanism, by which N-recognins select specific N-degrons (8). We compared the degradation and degradation pathways of CtPC-degrons with the sequence ELYKQI at different positions at or near the C terminus (Fig. 1*H*). The degron CtPC^ELYKQI^ was recognized by the SCF^Das1^-ligase, as were the shift variants ELYKQI-N, ELYKQI-YHS, ELYKQI-TIN, and ELYKQI-FRVI (Fig. 5*D*). Moreover, a varying impact of the *DAS1* deletion on the shift variants was observed, independent of the distance of ELYKQI from the C terminus. This is shown by the *das1Δ*/wildtype ratios: ELYKQI-TIN: 1.3; ELYKQI-N: 1.7; ELYKQI-YHS: 2.5; ELYKQI: 3.3; and ELYKQI-FRVI: 5.1 (Fig. 5*F* and Table S3). Thus, the varying impact of a *DAS1* deletion appear to result from different binding affinities depending on the last residues of the C terminus. Also, one C-degron variant (ELYKQI-FTCP) was not recognized by the SCF complex. It has been demonstrated in mammalian cells that the localization of specific amino acids at the extreme C terminus can be critical in the context of C-degrons and that the terminal carboxyl group can play a role in binding to a specific E3 ligase (14, 22, 23). Thus, the degradation pathways of the CtPC shift variants appear to rather depend on the amino acid composition of the last C-terminal residues than the difference in position of the ELYKQI sequence. These examples demonstrate the plasticity of C-terminal sequences and the difficulty to identify the molecular recognition pattern of specific sequences without high-resolution structure of the degron-recognizing protein. However, the ELYKQI pattern that is present also in all stable variants within the linker sequence between the LOV2 domain and the C-terminal peptide demonstrates that Das1 recognizes sequences at or close to the C terminus, and the same sequences are not recognized in the interior of the protein. Altogether, our data demonstrate that specific C-terminal residue pattern induces protein destabilization and that distinct PQC pathways monitor the C termini of proteins in yeast.

In general, the CtPC stability indices suggest that the specific positioning of certain amino acids is critical for protein stability. An assignment of more degron sequences to specific E3s is necessary to identify the specific recognition patterns for each E3. Several E3s were identified to detect uncommon C-terminal ends in proteins. As E3s might detect several amino acid patterns as aberrant that can be accomplished through different target-binding sites. A prime example for recognition of several degrons is Ubr1, the E3 targeting aberrant N termini in yeast (71). Three different substrate-binding sites have been identified in Ubr1, two of them bind distinct N-degrons and another one an internal degron. Obtaining a detailed structure of Das1 would help to understand the binding characteristics of this E3 better.

### Uncommon C termini create C-degrons

A possibility for the creation of uncommon C termini is a splicing or the translation of unspliced mRNA. A protection mechanism from the presence of a protein originating from the unspliced mRNA of *HAC1* that relies on degradation of this variant *via* SCF^Das1^ has been described (43). Interestingly, the mammalian counterpart of Hac1 named XBP-1 also shows increased proteolysis after translation of the unspliced XBP-1 mRNA (72). However, in this case, direct proteasomal recognition of the C terminus is involved followed by proteolysis. Alternative splicing was found to create a potent C-terminal encoded degron in case of the tumor suppressor MAX (73). These examples show that cellular observation of the C termini is important part of the PQC.

A second possibility for the creation of uncommon C termini are endoproteolytic events that cleave cellular proteins in two parts. Here, we tested the exposed C terminus of the N-terminal fragment of Scc1 after Esp1 proteolysis. Degradation of this fragment in the cellular context has been characterized (46, 47). The destabilizing features of the last six amino acids of the Scc1 C terminus suggest that Esp1 activity exposes a ubiquitin-dependent C-degron after Scc1 cleavage. This illustrates that the optogenetic approach offers a simple way to identify C-degrons and to investigate underlying degradation mechanisms independently of the cellular context. This is helpful for cases like Scc1, in which the degron gets released only after activation of Esp1 and in which elevated Scc1p fragment levels can be lethal in yeast (44).

A third possible mechanism to create alternative C termini is translational stop codon read-through. Read-through events in eukaryotic cells result in regulation of protein localization or stability by addition of a new C-terminal signal (42, 74, 75, 76). In human cells, translational stop-codon read-through of 20 genes is connected to heredity disorders because of lowered levels of the gene products. Extension of the proteins *via* stop-codon read-through leads to proteasomal degradation of these variants (77). A screen in *Caenorhabditis elegans* has revealed that accumulation of stop-codon read-through extended peptides is prevented by cotranslational and post-translational mechanisms that are also present in human cells (78). Our analysis suggests that stop-codon read-through may lead to appearance of C-degrons that induce proteolysis of the extended proteins. A similar mechanism has been suggested for the action of C-degrons in human cells (15). The importance of preventing erroneous stop codon read-through is indicated by the increased frequency of secondary stop codons at the positions +1, +2, and +3 in *S. cerevisiae* (79). In general, translational read-through depends on the identity of the stop codon, the sequence surrounding the stop codon, the level of mRNA, and the amounts of termination machinery components like eRF1 and eRF3 (80). It has been shown in yeast that mutational inactivation of eRF3 (in yeast Sup35) that leads to prion formation increases translational stop-codon read-through events (81). Protein quality pathways recognizing C-degrons originating from stop codon read-through events protect proteomes from accumulation of extended proteins that may have reduced functionality. Also, in the absence of deleterious events like the presence of Sup35-prions, translational stop codon read-through occurs. For translation of the Pde2-encoding mRNA, the read-through frequency has been determined to be 2.2% (42). Translational stop-codon read-through is quite common in a subset of highly expressed genes in budding yeast that in addition contain intrinsically disordered C termini (45, 82). Our analysis of first-frame extensions of highly expressed genes indicates that some of the resulting extended proteins are destabilized because of a disordered C terminus and the extension with a C-degron. Interestingly, the depletion of glycine at position −1 in natural proteins is conserved across eukaryotic proteomes, but it does not extend to proteins with uncommon C termini that are created by translational frameshift or read-through errors, nonsense-mediated mRNA decay transcripts, or small peptides encoded by upstream open reading frames in mammalian cells (24). It is remarkable that evolutionary pressure on the DNA sequence around the stop codon has opposing effects. At the end of the open reading frame, amino acids are encoded that generate stable proteins. After the stop codon, amino acids are favored that destabilize an extended protein. Thus, correctly formed proteins are in general stable, whereas extended proteins are destabilized by triggering PQC, which protects the cell from potentially deleterious effects of non-natural proteins. The importance of the latter mechanism is underscored by increased translational read-through events that are detectable in tumor tissues (83).

## Experimental procedures

### Yeast strains, growth conditions, and plasmids

The *S. cerevisiae* strains are derivatives of ESM356-1, BY4741, SUB62, MHY501, YYS40, and JD47-13C (48, 84, 85, 86, 87, 88). Strains are listed with their relevant genotypes in Table S1. Gene deletions were performed as described (89). Plasmids were transformed into yeast cells by the lithium acetate method (90). Blue light illumination (465 nm, 30 μmol m^−2^ s^−1^) of yeast cells was done using high-power LED strips (revoART), StraHat LED clusters (six clusters of 42 LEDs; revoART), or RGB LEDs (5050 RGP LEDS; revoART). Low florescence medium was used for incubation of yeast cells in blue light (33). Temperature-sensitive strains were grown at room temperature (25 °C) and shifted to the restrictive condition (37 °C) before harvesting: JD77 (*uba1*^ts^) strain and the corresponding wildtype JD47-13C were kept at restricted condition for 3 h, the TSA974 (*Cdc53*^ts^) strain and its wildtype BY4741 for 2 h, and the strains YGA1 (wildtype), YGA2 (*hsp110*^ts^), and YGA3 (*hsp70*^ts^) for 30 min. For usage of the galactose-inducible promoter, cells were grown in a preculture with 2% raffinose as C-source, and 2% galactose was added to the main culture. Plasmids are listed in Table S2. Random C-terminal sequences were inserted into a plasmid carrying the construct *sfGFP-P2A-mScarlett*^*I*^*-LOV2* (pSH59) using *in vivo* ligation in yeast and degenerated primers. All resulting C-terminal peptide sequences of the CtPCs are listed in Table S3.

### Cycloheximide chase assay, flow cytometer measurements, and fluorescence microscopy

For cycloheximide chases, cells were grown for 5 h in darkness, cycloheximide (200 μg/ml end concentration) was added, and the cells were incubated under blue light. Samples were taken every 20 min, sodium azide (10 μM end concentration) was added, and fluorescence was measured using a flow cytometer. Flow cytometer measurements were performed as described (25). Briefly, cells were diluted 1:10, transferred into a multi 96-well plate, and green and red fluorescence was measured in a flow cytometer (Attune NxT with an autosampler; Thermo Fisher). A blue laser (488 nm) was used to determine forward scatter (FSC) and sideward scatter, and a bandpass filter (530/30 nm) was used to measure green fluorescence. To detect red fluorescence, a yellow laser (561 nm) for excitation and a bandpass filter (620/15 nm) was used. Using the FSC (FSC-H blotted against FSC-A), the signals were gated on single cell events. Autofluorescence was determined by measuring yeast cells with an empty plasmid and used for background corrections. The data were analyzed using the program LibreOffice (The Document Foundation).

Microscopy of living yeast cells was done as previously described (91) with a Zeiss Axiovert 200 equipped with a Hamamatsu camera, enhanced GFP, and rhodamine filter sets, and a 63× Plan Apochromat oil lens (numerical aperture = 1.4) using the software Volocity 5.03 (PerkinElmer). Representative images were processed with ImageJ software (National Institutes of Health). All processing steps that involved changes to fluorescence intensity were performed in the same way to all images of the same fluorescence channel to preserve differences in fluorescence intensity.

### Immunoblotting, ubiquitylation assay, and quantitative imaging

Immunoblotting was performed as described (91) by alkaline lysis and trichloroacetic acid precipitation followed by SDS-PAGE and blotting. For signal detection, the antibodies mouse α-myc (Cell Signaling Technology), anti-TEV (kind gift of M. Ehrmann), and rabbit α-Tub1 (Abcam) were used in combination with the corresponding horseradish peroxidase-conjugated secondary antibody. The detection was done with a Chemostar imaging device (INTAS). Processing and quantification of the resulting image was carried out using ImageJ software (National Institutes of Health) as described (31).

For macroscopic quantitative imaging of yeast, colonies were replica plated onto two plates and incubated for 2 days in darkness or exposed to blue light (30 μmol m^−2^ s^−1^). Imaging and fluorescence detection was performed with the imager LAS4000 Fuji (GE Healthcare Life Sciences GmbH) using green epi lights (520 nm) and blue epi lights (460 nm). Resulting images were processed and quantified using the Fiji software (National Institutes of Health) (92).

### Bioinformatics analysis

Sequences of the yeast genome and yeast proteome were obtained from the *S. cerevisiae* database (93). Libre office calc (The Document Foundation) was used to obtain frequencies of C-terminal amino acids. Potential first-frame read-through peptides were obtained by translating the first-frame genomic sequence directly after a stop codon until the next stop codon with the tool EMBOSS transseq (94). Destabilizing and stabilizing C-terminal amino acid sequences were normalized to the frequency, with which a specific amino acid appears in the coding sequences of budding yeast genes. The frequencies for codon usage in *S. cerevisiae* were obtained from the *S. cerevisiae* genome database (yeastgenome.org). The online resource heatmapper (heatmapper.org) was used to generate visualizations of amino acid frequencies at the C terminus. Hydrophobicity of C-terminal amino acids was analyzed with an online GRAVY calculator (gravy-calculator.de).

## Data availability

All data are contained within the article.

## Supporting information

This article contains supporting information (45, 94, 95).

## Conflict of interest

The authors declare that they have no conflicts of interest with the contents of this article.
