# Supplementary material for: C-terminal sequence stability profiling in Saccharomyces cerevisiae reveals protective protein quality control pathways
Source: J Biol Chem. 2023 Aug 16;299(9):105166. doi: 10.1016/j.jbc.2023.105166 (PMC10493509; doi:10.1016/j.jbc.2023.105166)
Supplement: Supporting Table S1 [file mmc1.docx]

**Supplemental Table S1: Yeast Strains**

| **Name** | **Genotype** | **Source** |
| --- | --- | --- |
| ESM356-1 | *MATa ura3-53 leu2Δ1 his3Δ200 trp1Δ63* | (1) |
| JD47-13C | *MATa his3-Δ200 leu2-3,112 lys2-801 trp1-Δ63 ura3-52* | (2) |
| JD77 | JD47-13C *uba1Δ::HIS3 pRSts64-1(uba1-ts; TRP1)* | (2) |
| MHY501 | *MATα his3-Δ200 lys2-801 leu2-3,112 trp1-1 ura3-52* | (3) |
| MHY1631 | MHY501 *doa10Δ::HIS3* | (3) |
| Y02708 | BY4741 *hrt3Δ::kanMX4* | EUROSCARF |
| Y05277 | BY4741 *mdm30Δ::kanMX4* | EUROSCARF |
| Y03578 | *BY4741 mfb1Δ::kanMX4* | EUROSCARF |
| Y01376 | *BY4741 rtt101Δ::kanMX4* | EUROSCARF |
| Y01133 | *BY4741 skp2Δ::kanMX4* | EUROSCARF |
| Y00482 | *BY4741 ufo1Δ::kanMX4* | EUROSCARF |
| Y01276 | BY4741 *Yjl149w(Das1)Δ::kanMX4* | EUROSCARF |
| Y03665 | *BY4741 ydr306cΔ::kanMX4* | EUROSCARF |
| Y04173 | *BY4741 ylr224wΔ::kanMX4* | EUROSCARF |
| Y01856 | *BY4741 dia2Δ::kanMX4* | EUROSCARF |
| YCT1084 | ESM356-1 *Ubr1Δ::hphNT1* | (4) |
| YYS40 | *MATa leu2 his3 ura3 trp1 ade2 can1 ssd1 RPN11-3FLAG::HIS3* | (5) |
| YDAK47 | YYS40 *rpn1Δ::KanMX rpn13Δ::NatMX rpn10ΔUIM rpn1ΔT1::LEU2* (cen plasmid) *rpn13Δpru::URA3* (cen plasmid) | (5) |
| YDAK36 | YYS40 *rpn1Δ::NatMX rpn1ΔT1::LEU2 (*cen plasmid) | (5) |
| YCMH8 | YYS40 *rpn13Δ::NatMX rpn13Δpru::URA3* (cen plasmid) | (5) |
| YDAK34 | YYS40 *rpn10Δ::NatMX rpn10ΔUIM::LEU2* (cen plasmid) | (5) |
| YSH35 | YYS40 *rpn1Δ::KanMX rpn10Δ::NatMX rpn1ΔT1::LEU2* (cen plasmid) *rpn10ΔUIM::URA3* (cen plasmid) | This work |
| YNDN1 | *MATa RPN11-3FLAG::HIS3 rpn10∆::NatMX* | (5) |
| YNDN2 | *MATa RPN11-3FLAG::HIS3 rpn13∆::NatMX* | (5) |
| BY4741 | *MATa his3Δ1 leu2Δ0 met15Δ0 ura3Δ0* | (6) |
| Y04077 | BY4741 *san1Δ::KanMX* | EUROSCARF |
| YSH31 | BY4741 *doa10Δ::NatNT2 ubr1Δ::hphNT1 san1Δ::KanMX* | This work |
| SUB62 | *MATa his3-Δ200 lys2-801 leu2-3,112 trp1-1 ura3-52* | (7) |
| DY106 | SUB62 *rpt1^K256S^* | (7) |
| DY62 | SUB62 *rpt2^K229R S241F^* | (7) |
| DY93 | SUB62 *rpt3^K219R^* | (7) |
| DY219 | SUB62 *rpt4^K297R^* | (7) |
| DY155 | SUB62 *rpt5^K228R^* | (7) |
| DY100 | SUB62 *rpt6^K195R^* | (7) |
| WCG4a | *MATa ura3 leu2-3,112 his3-11,15 CanS Gal+* | (8) |
| WCG4a/11/22 | WCG4a *pre1-1 pre2-2* | (8) |
| TSA974 | BY4741 *cdc53-1::kanMX* | (9) |
| AM190 | JD47-13C *rpn5-ΔCT(W415STOP)::URA3* | (10) |
| YGA1 | BY4741 *MATa his3Δ1 leu2Δ0 PRE1FH::URA3* | (11) |
| YGA2 | YGA1 *sse1Δ::hphMX4 sse2Δ::kanMX4 his3Δ1::[his3Δ::hisG]-sse1-200* | (11) |
| YGA3 | YGA1 *ssa1-45 ssa2Δ::natMX4 ssa3Δ::hphMX4 ssa4Δ::kanMX4* | (11) |
| W303-1B | *MATα leu2-3,112 his3-11,15 ura3-1 trp1-1 ade2-1 can1-100* | (12) |
| CBO18 | *MATa leu2-3,112 his3-11,15 ura3-1 trp1-1 ade2-1 can1-100 pep4Δ::HIS3 prb1Δ::hisG prc1Δ::hisG* | (12) |

**Literature**

1. Pereira, G., Tanaka, T. U., Nasmyth, K., and Schiebel, E. (2001) Modes of spindle pole body inheritance and segregation of the Bfa1p-Bub2p checkpoint protein complex. *EMBO J.* **20**, 6359–6370

2. Palanimurugan, R., Scheel, H., Hofmann, K., and Jürgen Dohmen, R. (2004) Polyamines regulate their synthesis by inducing expression and blocking degradation of ODC antizyme. *EMBO J.* **23**, 4857–4867

3. Swanson, R., Locher, M., and Hochstrasser, M. (2001) A conserved ubiquitin ligase of the nuclear envelope / endoplasmic reticulum that functions in both ER-associated and Mat ␣ 2 repressor degradation. *Genes Dev.* **2**, 2660–2674

4. Taxis, C., Stier, G., Spadaccini, R., and Knop, M. (2009) Efficient protein depletion by genetically controlled deprotection of a dormant N-degron. *Mol. Syst. Biol.* **5**, 267

5. Cundiff, M. D., Hurley, C. M., Wong, J. D., Boscia, J. A., Bashyal, A., Rosenberg, J., Reichard, E. L., Nassif, N. D., Brodbelt, J. S., and Kraut, D. A. (2019) Ubiquitin receptors are required for substrate-mediated activation of the proteasome’s unfolding ability. *Sci. Reports 2019 91*. **9**, 1–17

6. Brachmann, C. B., Davies, a, Cost, G. J., Caputo, E., Li, J., Hieter, P., and Boeke, J. D. (1998) Designer deletion strains derived from Saccharomyces cerevisiae S288C: a useful set of strains and plasmids for PCR-mediated gene disruption and other applications. *Yeast*. **14**, 115–32

7. Rubin, D. M., Glickman, M. H., Larsen, C. N., Dhruvakumar, S., and Finley, D. (1998) Active site mutants in the six regulatory particle ATPases reveal multiple roles for ATP in the proteasome. *EMBO J.* **17**, 4909–4919

8. Heinemeyer, W., Gruhler, a, Möhrle, V., Mahé, Y., and Wolf, D. H. (1993) PRE2, highly homologous to the human major histocompatibility complex-linked RING10 gene, codes for a yeast proteasome subunit necessary for chrymotryptic activity and degradation of ubiquitinated proteins. *J. Biol. Chem.* **268**, 5115–20

9. Li, Z., Vizeacoumar, F. J., Bahr, S., Li, J., Warringer, J., Vizeacoumar, F. S., Min, R., Vandersluis, B., Bellay, J., Devit, M., Fleming, J. A., Stephens, A., Haase, J., Lin, Z. Y., Baryshnikova, A., Lu, H., Yan, Z., Jin, K., Barker, S., Datti, A., Giaever, G., Nislow, C., Bulawa, C., Myers, C. L., Costanzo, M., Gingras, A. C., Zhang, Z., Blomberg, A., Bloom, K., Andrews, B., and Boone, C. (2011) Systematic exploration of essential yeast gene function with temperature-sensitive mutants. *Nat. Biotechnol. 2011 294*. **29**, 361–367

10. Gödderz, D., Schäfer, E., Palanimurugan, R., and Dohmen, R. J. (2011) The N-terminal unstructured domain of yeast ODC functions as a transplantable and replaceable ubiquitin-independent degron. *J. Mol. Biol.* **407**, 354–67

11. Kandasamy, G., and Andréasson, C. (2018) Hsp70–Hsp110 chaperones deliver ubiquitin-dependent and -independent substrates to the 26S proteasome for proteolysis in yeast. *J. Cell Sci.* **131**, jcs210948

12. Graham, T. R., and Emr, S. D. (1991) Compartmental organization of Golgi-specific protein modification and vacuolar protein sorting events defined in a yeast sec18 (NSF) mutant. *J. Cell Biol.* **114**, 207–18
