# Supplementary material for: C-terminal sequence stability profiling in Saccharomyces cerevisiae reveals protective protein quality control pathways
Source: J Biol Chem. 2023 Aug 16;299(9):105166. doi: 10.1016/j.jbc.2023.105166 (PMC10493509; doi:10.1016/j.jbc.2023.105166)
Supplement: Supporting Table S2 [file mmc2.docx]

**Supplemental Table S2: Plasmids**

| **Name** | **Features** | **Source** |
| --- | --- | --- |
| pRS314 | *TRP1 ARS209/CEN2 ori bla* | (Sikorski and Hieter, 1989) |
| pRS315 | *LEU2 ARS209/CEN2 ori bla* | (Sikorski and Hieter, 1989) |
| pRS316 | *URA3 ARS209/CEN2 ori bla* | (Sikorski and Hieter, 1989) |
| pCT260 | pRS316 P*_GAL1_-p14D^122Y^-TEV ^T17D I77V S219V^* | (Taxis et al., 2009) |
| pSH109 | pRSS316 P*_gal1_-p14D122Y-TEV ^T17D I77V S219V^-ENLYFQG-CtPC^AEAKCL^* | This work |
| pSH110 | pRSS316 P*_gal1_-p14D122Y-TEV ^T17D I77V S219V^-ENLYFQP-CtPC^AEAKCL^* | This work |
| pSH112 | pRSS316 P*_gal1_-p14D122Y-TEV ^T17D I77V S219V^-ENLYFQP-CtPC^AEAKVM^* | This work |
| pSH139 | pRSS316 P*_gal1_-p14D122Y-TEV ^T17D I77V S219V^-ENLYFQP-CtPC^VVRFKW^* | This work |
| pSH140 | pRSS316 P*_gal1_-p14D122Y-TEV ^T17D I77V S219V^-ENLYFQP-CtPC^AEAKES^* | This work |
| pSH141 | pRSS316 P*_gal1_-p14D122Y-TEV ^T17D I77V S219V^-ENLYFQP-CtPC^AEAKLF^* | This work |
| pSH59 | pRS315 P*_ADH1_-sfGFP-P2A-mScarlett-i-iLID^Al46Δ^* | This work |
| pDS350 | pRS315 P*_ADH1_-sfGFP-P2A-mScarlett-i-iLID^Al46Δ^-CtPC^AEAKEL^* | This work |
| pDS351 | pRS315 P*_ADH1_-sfGFP-P2A-mScarlett-i-iLID^Al46Δ^-CtPC^AECKEL^* | This work |
| pDS352 | pRS315 P*_ADH1_-sfGFP-P2A-mScarlett-i-iLID^Al46Δ^-CtPC^AEACEL^* | This work |
| pDS353 | pRS315 P*_ADH1_-sfGFP-P2A-mScarlett-i-iLID^Al46Δ^-CtPC^AEAKCL^* | This work |
| pDS354 | pRS315 P*_ADH1_-sfGFP-P2A-mScarlett-i-iLID^Al46Δ^-CtPC^AEAKEC^* | This work |
| pDS355 | pRS315 P*_ADH1_-sfGFP-P2A-mScarlett-i-iLID^Al46Δ^-CtPC^AEWKEL^* | This work |
| pDS356 | pRS315 P*_ADH1_-sfGFP-P2A-mScarlett-i-iLID^Al46Δ^-CtPC^AEAWEL^* | This work |
| pDS357 | pRS315 P*_ADH1_-sfGFP-P2A-mScarlett-i-iLID^Al46Δ^-CtPC^AEAKWL^* | This work |
| pSH77 | pRS315 P*_ADH1_-sfGFP-P2A-mScarlett-i-iLID^Al46Δ^-CtPC^KQIYHS^* | This work |
| pSH78 | pRS315 P*_ADH1_-sfGFP-P2A-mScarlett-i-iLID^Al46Δ^-CtPC^VVRFKW^* | This work |
| pSH79 | pRS315 P*_ADH1_-sfGFP-P2A-mScarlett-i-iLID^Al46Δ^-CtPC*^AEAKSH^ | This work |
| pSH80 | pRS315 P*_ADH1_-sfGFP-P2A-mScarlett-i-iLID^Al46Δ^-CtPC^AEAKSM^* | This work |
| pSH81 | pRS315 P*_ADH1_-sfGFP-P2A-mScarlett-i-iLID^Al46Δ^-CtPC^AEAKAN^* | This work |
| pSH82 | pRS315 P*_ADH1_-sfGFP-P2A-mScarlett-i-iLID^Al46Δ^-CtPC^AEAKAY^* | This work |
| pSH83 | pRS315 P*_ADH1_-sfGFP-P2A-mScarlett-i-iLID^Al46Δ^-CtPC^AEAKAF^* | This work |
| pSH84 | pRS315 P*_ADH1_-sfGFP-P2A-mScarlett-i-iLID^Al46Δ^-CtPC^AEAKSI^* | This work |
| pSH85 | pRS315 P*_ADH1_-sfGFP-P2A-mScarlett-i-iLID^Al46Δ^-CtPC^SVFSPG^* | This work |
| pSH86 | pRS315 P*_ADH1_-sfGFP-P2A-mScarlett-i-iLID^Al46Δ^-CtPC^AEAKTH^* | This work |
| pSH88 | pRS315 P*_ADH1_-sfGFP-P2A-mScarlett-i-iLID^Al46Δ^-CtPC*^AEAKVY^ | This work |
| pSH89 | pRS315 P*_ADH1_-sfGFP-P2A-mScarlett-i-iLID^Al46Δ^-CtPC ^KQIITA^* | This work |
| pSH90 | pRS315 P*_ADH1_-sfGFP-P2A-mScarlett-i-iLID^Al46Δ^-CtPC^AEAKIY^* | This work |
| pSH91 | pRS315 P*_ADH1_-sfGFP-P2A-mScarlett-i-iLID^Al46Δ^-CtPC^LKLSRI^* | This work |
| pSH92 | pRS315 P*_ADH1_-sfGFP-P2A-mScarlett-i-iLID^Al46Δ^-CtPC^KAAGVG^* | This work |
| pSH93 | pRS315 P*_ADH1_-sfGFP-P2A-mScarlett-i-iLID^Al46Δ^-CtPC^GVIELV^* | This work |
| pSH94 | pRS315 P*_ADH1_-sfGFP-P2A-mScarlett-i-iLID^Al46Δ^-CtPC^AEAKVG^* | This work |
| pSH95 | pRS315 P*_ADH1_-sfGFP-P2A-mScarlett-i-iLID^Al46Δ^-CtPC^AEAKFL^* | This work |
| pSH96 | pRS315 P*_ADH1_-sfGFP-P2A-mScarlett-i-iLID^Al46Δ^-CtPC^AEAKDP^* | This work |
| pSH97 | pRS315 P*_ADH1_-sfGFP-P2A-mScarlett-i-iLID^Al46Δ^-CtPC^AEAKVM^* | This work |
| pSH98 | pRS315 P*_ADH1_-sfGFP-P2A-mScarlett-i-iLID^Al46Δ^-CtPC^AEAKVR^* | This work |
| pSH99 | pRS315 P*_ADH1_-sfGFP-P2A-mScarlett-i-iLID^Al46Δ^-CtPC^AEAKVL^* | This work |
| pSH100 | pRS315 P*_ADH1_-sfGFP-P2A-mScarlett-i-iLID^Al46Δ^-CtPC^AEAKAI^* | This work |
| pSH101 | pRS315 P*_ADH1_-sfGFP-P2A-mScarlett-i-iLID^Al46Δ^-CtPC^AEAKIC^* | This work |
| pSH102 | pRS315 P*_ADH1_-sfGFP-P2A-mScarlett-i-iLID^Al46Δ^-CtPC^AEAKAQ^* | This work |
| pSH103 | pRS315 P*_ADH1_-sfGFP-P2A-mScarlett-i-iLID^Al46Δ^-CtPC^DLGERTL^* | This work |
| pSH104 | pRS315 P*_ADH1_-sfGFP-P2A-mScarlett-i-iLID^Al46Δ^-CtPC^AEAKTL^* | This work |
| pSH105 | pRS315 P*_ADH1_-sfGFP-P2A-mScarlett-i-iLID^Al46Δ^-CtPC^LLVRRR^* | This work |
| pSH106 | pRS315 P*_ADH1_-sfGFP-P2A-mScarlett-i-iLID^Al46Δ^-CtPC^AEAKLF^* | This work |
| pSH107 | pRS314 P*_ADH1_-sfGFP-P2A-mScarlett-i-iLID^Al46Δ^-CtPC^AEAKAY^* | This work |
| pSH108 | pRS314 P*_ADH1_-sfGFP-P2A-mScarlett-i-iLID^Al46Δ^-CtPC^AEAKAN^* | This work |
| pSH111 | pRSS316 *P_gal1_-p14D122Y-TEV^T17D I77V S219V^-ENLYFQG-CtPC^AEAKVM^* | This work |
| pSH113 | pRSS316 *P_gal1_-p14D122Y-TEV^T17D I77V S219V^-ENLYFQG-CtPC^AEAKVM^* | This work |
| pSH114 | pRS316 P*_gal1_-p14D122Y-TEV^T17D I77V S219V^-ENLYFQG-CtPC^VVRFKW^* | This work |
| pSH115 | pRS315 P*_ADH1_-sfGFP-P2A-mScarlett-i-iLID^Al46Δ^-CtPC*^AEASAA^ | This work |
| pSH116 | pRS315 P*_ADH1_-sfGFP-P2A-mScarlett-i-iLID^Al46Δ^-CtPC^AEASYS^* | This work |
| pSH117 | pRS315 P*_ADH1_-sfGFP-P2A-mScarlett-i-iLID^Al46Δ^-CtPC*^AEANPA^ | This work |
| pSH118 | pRS315 P*_ADH1_-sfGFP-P2A-mScarlett-i-iLID^Al46Δ^-CtPC^AEAYVA^* | This work |
| pSH119 | pRS315 P*_ADH1_-sfGFP-P2A-mScarlett-i-iLID^Al46Δ^-CtPC*^AEACQV^ | This work |
| pSH120 | pRS315 P*_ADH1_-sfGFP-P2A-mScarlett-i-iLID^Al46Δ^-CtPC^AEATLV^* | This work |
| pSH121 | pRS315 P*_ADH1_-sfGFP-P2A-mScarlett-i-iLID^Al46Δ^-CtPC^QI^*^AEAR^ | This work |
| pSH122 | pRS315 P*_ADH1_-sfGFP-P2A-mScarlett-i-iLID^Al46Δ^-CtPC^AEAWAR^* | This work |
| pSH123 | pRS315 P*_ADH1_-sfGFP-P2A-mScarlett-i-iLID^Al46Δ^-CtPC^CKRLTC^* | This work |
| pSH124 | pRS315 P*_ADH1_-sfGFP-P2A-mScarlett-i-iLID^Al46Δ^-CtPC^QIFRVI^* | This work |
| pSH125 | pRS315 P*_ADH1_-sfGFP-P2A-mScarlett-i-iLID^Al46Δ^-CtPC^AVLAKG^* | This work |
| pSH126 | pRS315 P*_ADH1_-sfGFP-P2A-mScarlett-i-iLID^Al46Δ^-CtPC^ELYKQI^* | This work |
| pSH127 | pRS315 P*_ADH1_-sfGFP-P2A-mScarlett-i-iLID^Al46Δ^-CtPC^QIFTCP^* | This work |
| pSH128 | pRS315 P*_ADH1_-sfGFP-P2A-mScarlett-i-iLID^Al46Δ^-CtPC^LYKQIN^* | This work |
| pSH129 | pRS315 P*_ADH1_-sfGFP-P2A-mScarlett-i-iLID^Al46Δ^-CtPC^LFVAEK^* | This work |
| pSH130 | pRS315 P*_ADH1_-sfGFP-P2A-mScarlett-i-iLID^Al46Δ^-CtPC^LLESCN^* | This work |
| pSH131 | pRS315 P*_ADH1_-sfGFP-P2A-mScarlett-i-iLID^Al46Δ^-CtPC^MVGFSE^* | This work |
| pSH132 | pRS315 P*_ADH1_-sfGFP-P2A-mScarlett-i-iLID^Al46Δ^-CtPC^AEARKL^* | This work |
| pSH133 | pRS315 P*_ADH1_-sfGFP-P2A-mScarlett-i-iLID^Al46Δ^-CtPC^AEALPY^* | This work |
| pSH134 | pRS315 P*_ADH1_-sfGFP-P2A-mScarlett-i-iLID^Al46Δ^-CtPC^AEAKTS^* | This work |
| pSH135 | pRS315 P*_ADH1_-sfGFP-P2A-mScarlett-i-iLID^Al46Δ^-CtPC^AEASRG^* | This work |
| pSH136 | pRS315 P*_ADH1_-sfGFP-P2A-mScarlett-i-iLID^Al46Δ^-CtPC^AEARLV^* | This work |
| pSH137 | pRS315 P*_ADH1_-sfGFP-P2A-mScarlett-i-iLID^Al46Δ^-CtPC^AEAEMA^* | This work |
| pSH138 | pRS315 P*_ADH1_-sfGFP-P2A-mScarlett-i-iLID^Al46Δ^-CtPC^SLEVGR^* | This work |
| pSH142 | pRS315 P*_ADH1_-sfGFP-P2A-mScarlett-i-iLID^Al46Δ^-CtPC^PITSLF^* | This work |
| pSH143 | pRS315 P*_ADH1_-sfGFP-P2A-mScarlett-i-iLID^Al46Δ^-CtPC^RAYRCS^* | This work |
| pSH144 | pRS315 P*_ADH1_-sfGFP-P2A-mScarlett-i-iLID^Al46Δ^-CtPC^PTILIS^* | This work |
| pSH145 | pRS315 P*_ADH1_-sfGFP-P2A-mScarlett-i-iLID^Al46Δ^-CtPC^AEALAY^* | This work |
| pSH146 | pRS315 P*_ADH1_-sfGFP-P2A-mScarlett-i-iLID^Al46Δ^-CtPC^RSALIW^* | This work |
| pSH147 | pRS315 P*_ADH1_-sfGFP-P2A-mScarlett-i-iLID^Al46Δ^-CtPC^TLTRVM^* | This work |
| pSH148 | pRS315 P*_ADH1_-sfGFP-P2A-mScarlett-i-iLID^Al46Δ^-CtPC^GTKKMI^* | This work |
| pSH149 | pRS315 P*_ADH1_-sfGFP-P2A-mScarlett-i-iLID^Al46Δ^-CtPC^MRVMIR^* | This work |
| pSH150 | pRS315 P*_ADH1_-sfGFP-P2A-mScarlett-i-iLID^Al46Δ^-CtPC^TIDCAE^* | This work |
| pSH151 | pRS315 P*_ADH1_-sfGFP-P2A-mScarlett-i-iLID^Al46Δ^-CtPC^QKITIN^* | This work |
| pSH152 | pRS315 P*_ADH1_-sfGFP-P2A-mScarlett-i-iLID^Al46Δ^-CtPC^LYKQIFV^* | This work |
| pSH153 | pRS315 P*_ADH1_-sfGFP-P2A-mScarlett-i-iLID^Al46Δ^-CtPC^FDIRKT^* | This work |
| pSH154 | pRS315 P*_ADH1_-sfGFP-P2A-mScarlett-i-iLID^Al46Δ^-CtPC^LFMTMD^* | This work |
| pSH161 | pRS315 P*_ADH1_-sfGFP-P2A-mScarlett-i-iLID^Al46Δ^-CtPC^AEAKEL^* | This work |
| pSH162 | pRS315 P*_ADH1_-sfGFP-P2A-mScarlett-i-iLID^Al46Δ^-CtPC^AEAKPM^* | This work |
| pSH164 | pRS315 P*_ADH1_-sfGFP-P2A-mScarlett-i-iLID^Al46Δ^-CtPC^AEAKPT^* | This work |
| pSH165 | pRS315 P*_ADH1_-sfGFP-P2A-mScarlett-i-iLID^Al46Δ^-CtPC^AEAKYQ^* | This work |
| pSH166 | pRS315 P*_ADH1_-sfGFP-P2A-mScarlett-i-iLID^Al46Δ^-CtPC^AEAKDS^* | This work |
| pSH167 | pRS315 P*_ADH1_-sfGFP-P2A-mScarlett-i-iLID^Al46Δ^-CtPC^AEAKDS^* | This work |
| pSH168 | pRS315 P*_ADH1_-sfGFP-P2A-mScarlett-i-iLID^Al46Δ^-CtPC^IAEAKP^* | This work |
| pSH169 | pRS315 P*_ADH1_-sfGFP-P2A-mScarlett-i-iLID^Al46Δ^-CtPC^AEAKVE^* | This work |
| pSH170 | pRS315 P*_ADH1_-sfGFP-P2A-mScarlett-i-iLID^Al46Δ^-CtPC^AEAKYY^* | This work |
| pSH171 | pRS315 P*_ADH1_-sfGFP-P2A-mScarlett-i-iLID^Al46Δ^-CtPC^AEAKEL^* | This work |
| pSH172 | pRS315 P*_ADH1_-sfGFP-P2A-mScarlett-i-iLID^Al46Δ^-CtPC^AEAKWH^* | This work |
| pSH174 | pRS315 P*_ADH1_-sfGFP-P2A-mScarlett-i-iLID^Al46Δ^-CtPC^AEAKFT^* | This work |
| pSH175 | pRS315 P*_ADH1_-sfGFP-P2A-mScarlett-i-iLID^Al46Δ^-CtPC^AEAKEC^* | This work |
| pSH176 | pRS315 P*_ADH1_-sfGFP-P2A-mScarlett-i-iLID^Al46Δ^-CtPC^AEAKDP^* | This work |
| pSH177 | pRS315 P*_ADH1_-sfGFP-P2A-mScarlett-i-iLID^Al46Δ^-CtPC^AEAKWM^* | This work |
| pSH178 | pRS315 P*_ADH1_-sfGFP-P2A-mScarlett-i-iLID^Al46Δ^-CtPC^AEAKRF^* | This work |
| pSH179 | pRS315 P*_ADH1_-sfGFP-P2A-mScarlett-i-iLID^Al46Δ^-CtPC^AEASAI^* | This work |
| pSH182 | pRS315 P*_ADH1_-sfGFP-P2A-mScarlett-i-iLID^Al46Δ^-CtPC^AEAKIY^* | This work |
| pSH183 | pRS315 P*_ADH1_-sfGFP-P2A-mScarlett-i-iLID^Al46Δ^-CtPC^KQIAEA^* | This work |
| pSH184 | pRS315 P*_ADH1_-sfGFP-P2A-mScarlett-i-iLID^Al46Δ^-CtPC^QIAEAL^* | This work |
| pSH185 | pRS315 P*_ADH1_-sfGFP-P2A-mScarlett-i-iLID^Al46Δ^-CtPC^TQATNQ^* | This work |
| pSH186 | pRS315 P*_ADH1_-sfGFP-P2A-mScarlett-i-iLID^Al46Δ^-CtPC^TNETGI^* | This work |
| pSH188 | pRS315 P*_ADH1_-sfGFP-P2A-mScarlett-i-iLID^Al46Δ^-CtPC^AEAKEL^* | This work |
| pSH189 | pRS315 P*_ADH1_-sfGFP-P2A-mScarlett-i-iLID^Al46Δ^-CtPC^AEATRL^* | This work |
| pSH190 | pRS315 P*_ADH1_-sfGFP-P2A-mScarlett-i-iLID^Al46Δ^-CtPC^AEALRM^* | This work |
| pSH191 | pRS315 P*_ADH1_-sfGFP-P2A-mScarlett-i-iLID^Al46Δ^-CtPC^AEASKK^* | This work |
| pSH192 | pRS315 P*_ADH1_-sfGFP-P2A-mScarlett-i-iLID^Al46Δ^-CtPC^AEAIWD^* | This work |
| pSH193 | pRS315 P*_ADH1_-sfGFP-P2A-mScarlett-i-iLID^Al46Δ^-CtPC ^AEADIQ^* | This work |
| pSH195 | pRS315 P*_ADH1_-sfGFP-P2A-mScarlett-i-iLID^Al46Δ^-CtPC^AEALCL^* | This work |
| pSH196 | pRS315 P*_ADH1_-sfGFP-P2A-mScarlett-i-iLID^Al46Δ^-CtPC^LDGPPV^* | This work |
| pSH197 | pRS315 P*_ADH1_-sfGFP-P2A-mScarlett-i-iLID^Al46Δ^-CtPC^TSHASP^* | This work |
| pSH198 | pRS315 P*_ADH1_-sfGFP-P2A-mScarlett-i-iLID^Al46Δ^-CtPC^LPYGAD^* | This work |
| pSH199 | pRS315 P*_ADH1_-sfGFP-P2A-mScarlett-i-iLID^Al46Δ^-CtPC^QSTQQ^* | This work |
| pSH200 | pRS315 P*_ADH1_-sfGFP-P2A-mScarlett-i-iLID^Al46Δ^-CtPC^NATTTTL^* | This work |
| pSH202 | pRS315 P*_ADH1_-sfGFP-P2A-mScarlett-i-iLID^Al46Δ^-CtPC^AEAQLH^* | This work |
| pSH203 | pRS315 P*_ADH1_-sfGFP-P2A-mScarlett-i-iLID^Al46Δ^-CtPC ^AEARII^* | This work |
| pSH204 | pRS315 P*_ADH1_-sfGFP-P2A-mScarlett-i-iLID^Al46Δ^-CtPC^AEACGL^* | This work |
| pSH206 | pRS315 P*_ADH1_-sfGFP-P2A-mScarlett-i-iLID^Al46Δ^-CtPC^QIAEAK^* | This work |
| pSH207 | pRS315 P*_ADH1_-sfGFP-P2A-mScarlett-i-iLID^Al46Δ^-CtPC^AEAKAD^* | This work |
| pSH211 | pRS315 P*_ADH1_-ssfGFP-P2A-mScarlett-i-iLID^Al46Δ^-CtPC^AEANVA^* | This work |
| pSH212 | pRS315 P*_ADH1_-ssfGFP-P2A-mScarlett-i-iLID^Al46Δ^-CtPC^FLASLT^* | This work |
| pSH138 | pRS315 P*_ADH1_-ssfGFP-P2A-mScarlett-i-iLID^Al46Δ^-Cdeg^SLEVGR^* | This work |
| pSH156 | pRS315 P*_ADH1_-ssfGFP-P2A-mScarlett-i-iLID^Al46Δ^-Cdeg^NIYIYY^* | This work |
| pSH158 | pRS315 P*_ADH1_-sfGFP-P2A-mCherry-3myc-iLID^Al46Δ^-CtPC^AEAKLF^* | This work |
| pSH157 | pRS315 P*_ADH1_-sfGFP-P2A-mCherry-3myc-iLID^Al46Δ^-CtPC^LLVRRR^* | This work |
| pDS358 | pRS315 P*_ADH1_-sfGFP-P2A-mScarlett-i-iLID^Al46Δ^-CtPC^AEAKEW^* | This work |
| pSH71 | pRS314 P*_ADH1_-sfGFP-P2A-mScarlett-i-iLID^Al46Δ^-CtPC^AEAKCL^* | This work |
| pSH107 | pRS314 P*_ADH1_-sfGFP-P2A-mScarlett-i-iLID^Al46Δ^-CtPC^AEAKAY^* | This work |
| pSH108 | pRS314 P*_ADH1_-sfGFP-P2A-mScarlett-i-iLID^Al46Δ^-CtPC^AEAKAN^* | This work |
| pSH159 | pRS314 P*_ADH1_-sfGFP-P2A-mScarlett-i-iLID^Al46Δ^-CtPC^AEAKTH^* | This work |
| pSH213 | pRS315 P*_ADH1_-sfGFP-P2A-mScarlett-i-iLID^Al46Δ^-CtPC^LWSEEL^* | This work |
| pSH215 | pRS315 P*_ADH1_-sfGFP-P2A-mScarlett-i-iLID^Al46Δ^-CtPC^AEADQT^* | This work |

Literature

Sikorski RS, Hieter P. 1989. A system of shuttle vectors and yeast host strains designed for efficient manipulation of DNA in Saccharomyces cerevisiae. *Genetics* **122**:19–27. doi:0378111995000377 [pii]

Taxis C, Stier G, Spadaccini R, Knop M. 2009. Efficient protein depletion by genetically controlled deprotection of a dormant N-degron. *Mol Syst Biol* **5**:267. doi:10.1038/msb.2009.25
