# Supplementary material for: C-terminal sequence stability profiling in Saccharomyces cerevisiae reveals protective protein quality control pathways
Source: J Biol Chem. 2023 Aug 16;299(9):105166. doi: 10.1016/j.jbc.2023.105166 (PMC10493509; doi:10.1016/j.jbc.2023.105166)
Supplement: Supporting Figures S1–S14 [file mmc4.docx]

**Supporting information**

**C-terminal sequence stability profiling in *Saccharomyces cerevisiae* reveals protective protein quality control pathways**

**Sophia Hasenjäger, Andrea Bologna, Lars-Oliver Essen, Roberta Spadaccini, and Christof Taxis**

**
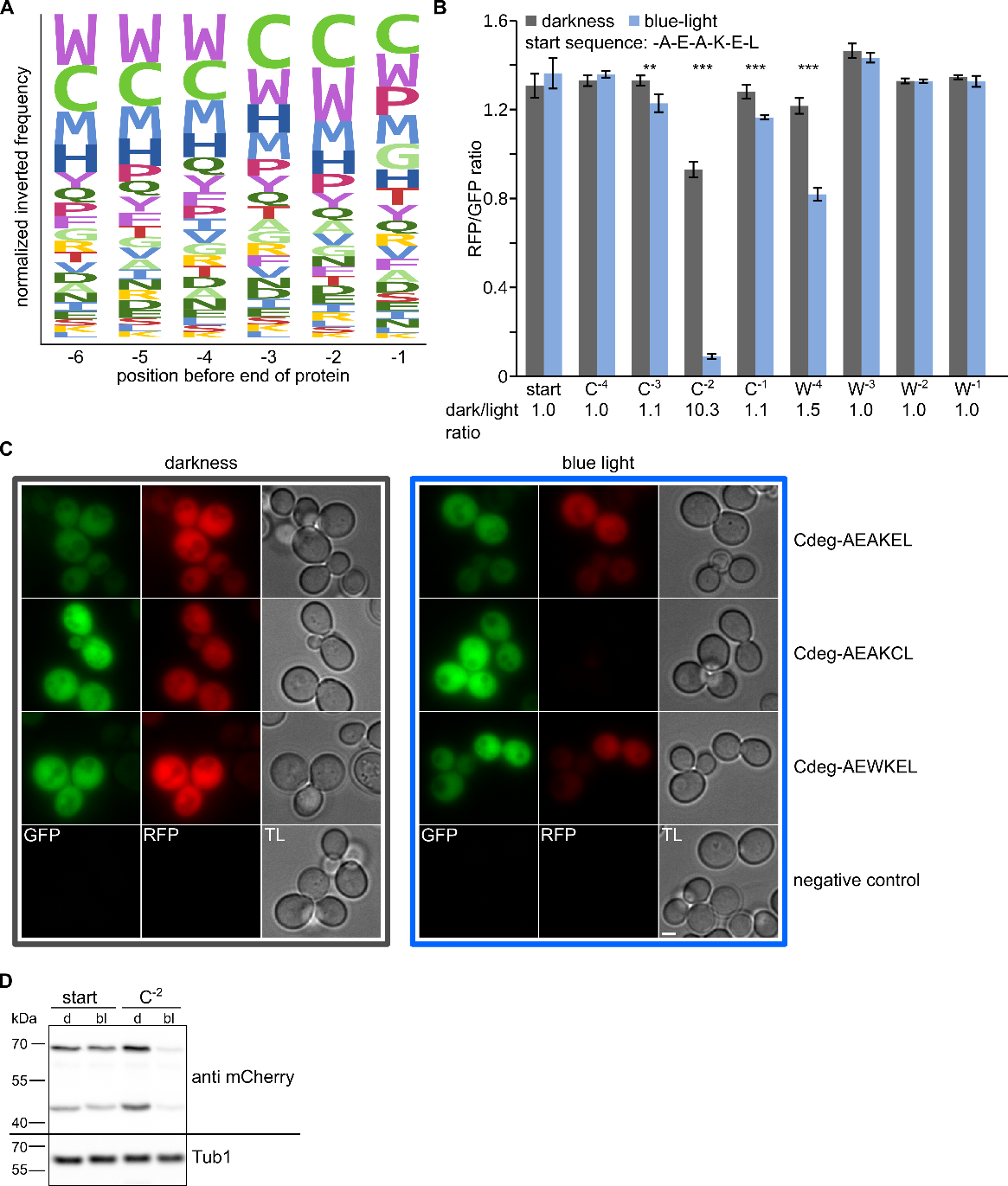
**

**Supplementary Figure S1: Usage of an optogenetic switch fused to a red-fluorescence reporter for the targeted identification of destabilizing C-terminal amino acids.** A) Plot of the normalized inverted frequency of amino acids at the last six position in the proteome of *S. cerevisiae*. (B) Ratio of RFP/GFP fluorescence intensities measured after 5 h incubation of yeast (strain ESM356-1) in blue light (30 μmol*m^-2^*s^-1^) and darkness, respectively. Targeted single exchanges are shown within the last six positions of a stable reporter with rare amino acids at the specific positions. The error bars show the standard error of the mean (n=4); A Students t-test was used to evaluate significance (***p*<0.01; ****p*<0.001). (C) The fluorescence intensity of cells carrying the indicated sfGFP-P2A-mScarlett-i-iLID^A416Δ^-Cdeg variants were recorded after 5 h of incubation in the dark or in blue light. Shown are intensity correlated RFP and GFP fluorescence images of the cells. Bar size: 2 µm. (D) Immunodetection of AEAKEL (starting sequence) and AEAKCL (C^-2^) constructs in ESM356-1 using αmCherry-antibody after 5 h incubation in the dark or in blue light.

**
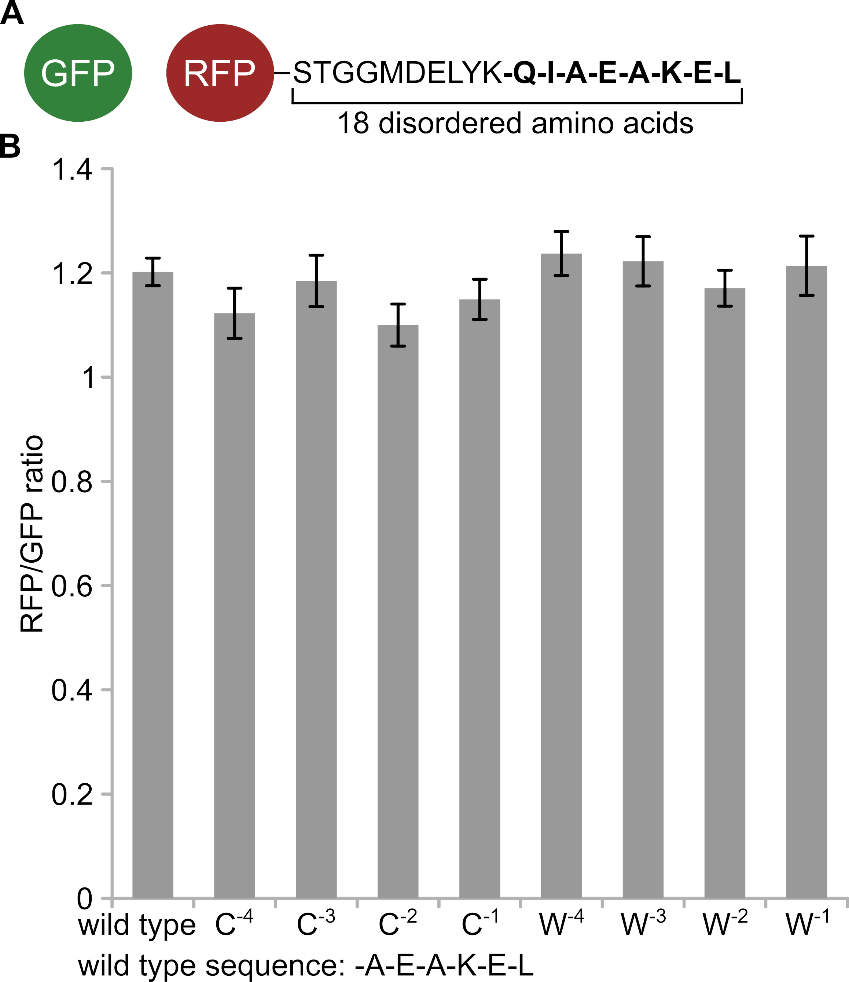
**

**Supplementary Figure S2: Usage of a short unstructured linker between RFP reporter and C-terminal sequences.** RFP/GFP fluorescence ratios of the split tandem fluorescent protein reporter GFP-P2A-mScarlett^I^ with an 18 amino acid long unstructured C-terminus. Targeted exchanges (same as in Supplementary Figure S1B) within the C-terminal sequence did not affect the fluorescence ratios significantly. Error bars: SEM.

**
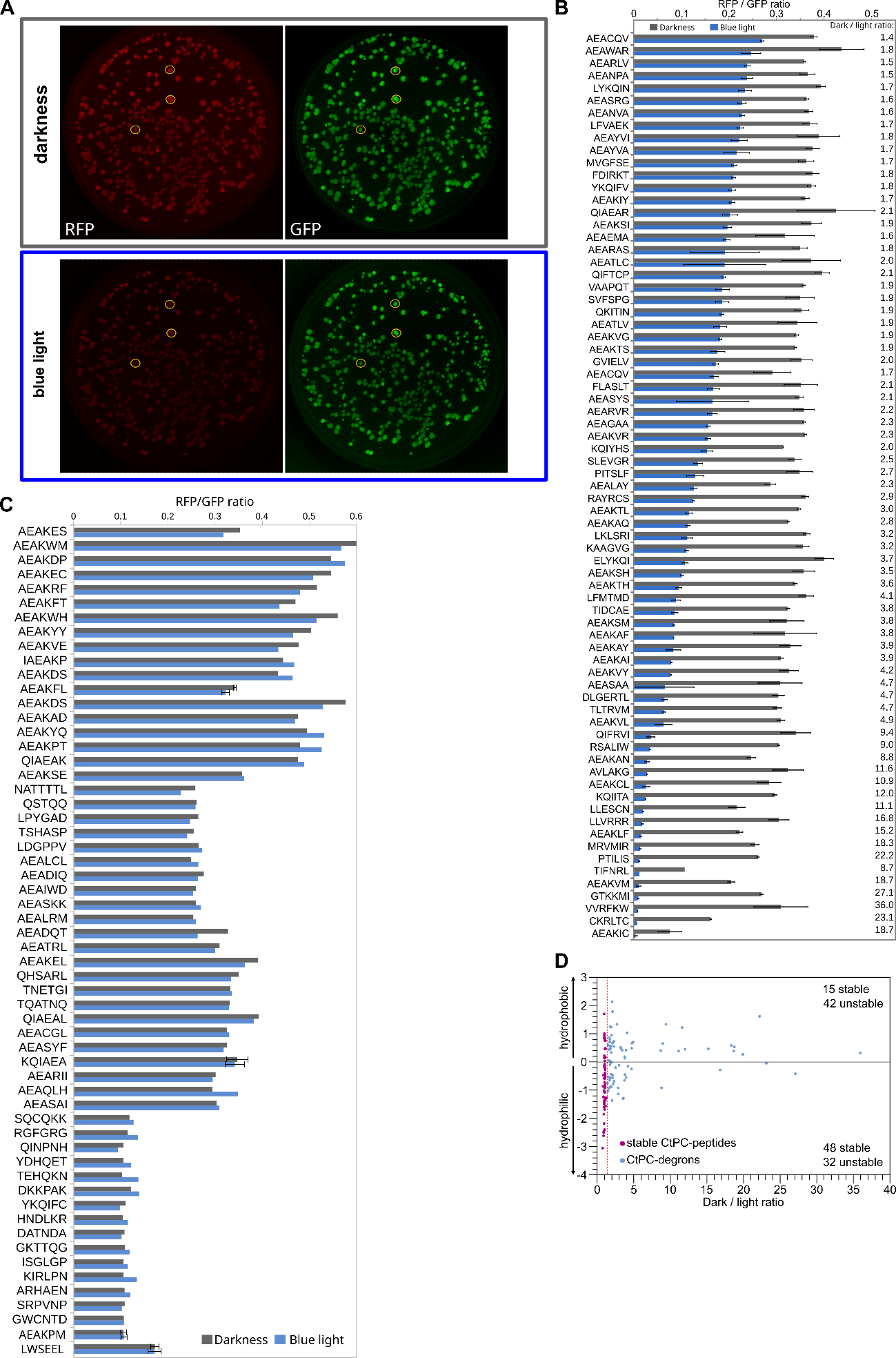
**

**Supplementary Figure S3: Generation of the C-terminal Peptide Collection (CtPC).** (A) Screening of RFP and GFP fluorescence of random C-termini with the optogenetic split-tandem fluorescent protein reporter. Yeast clones were grown on solid selective medium and duplicated. The duplicates were incubated under blue light and in darkness for two days, followed by fluorescence imaging of RFP and GFP fluorescence**.** (B) Ratio of RFP/GFP fluorescence intensities measured after 5 h incubation of wild type cells (ESM356-1) containing a CtPC plasmid in blue light (30 μmol*m^-2^*s^-1^) or darkness. All randomly generated CtPC variants are shown that have a dark/light switching ratio of 1.4 or more. Error bars: standard deviation (n=3). (C) Same as B for CtPC-variants with switching ratios of less than 1.4. The error bars show the standard deviations. (D) Dark/light switching ratios of CtPC variants plotted against the grand average of hydrophathy (GRAVY) analysis of the C-termini.


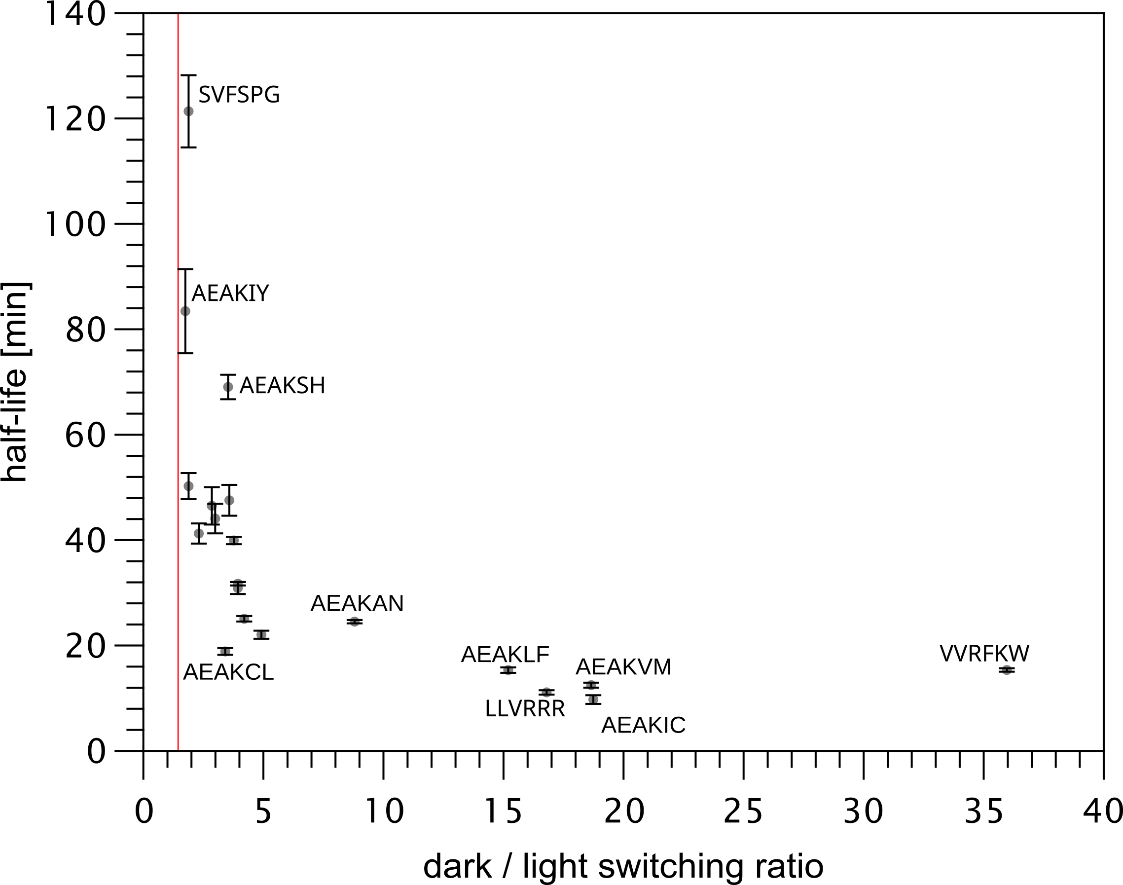


**Supplementary Figure S4:** Correlation of RFP reporter half-lives of different CtPC-degrons under blue light and the corresponding steady-state switching ratios between light conditions.

**
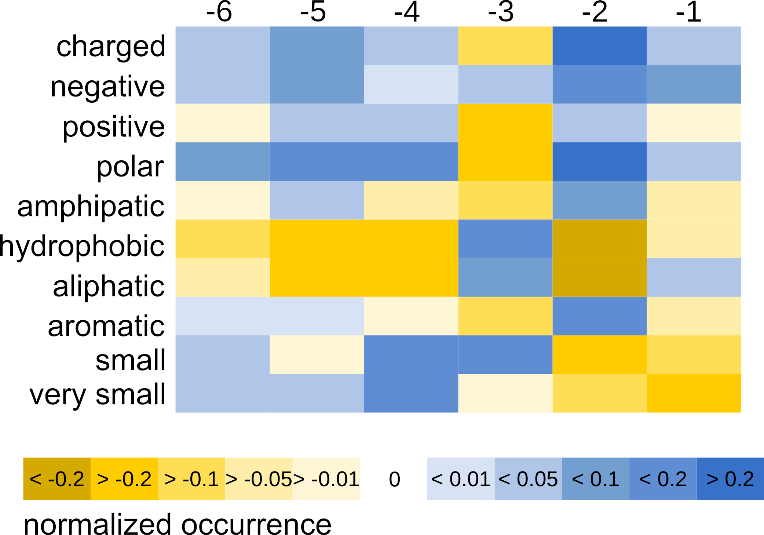
**

**Supplementary Figure S5: Amino acid properties in CtPC-degrons and stable CtPC variants.** Occurrence of amino acids in destabilized CtPC variants normalized to the occurrence of amino acids in stable CtPC variants. Values from -0.01 and lower (shades of yellow) indicate a frequent occurrence in destabilized variants and values from 0.01 and above (shades of blue) in stable variants.


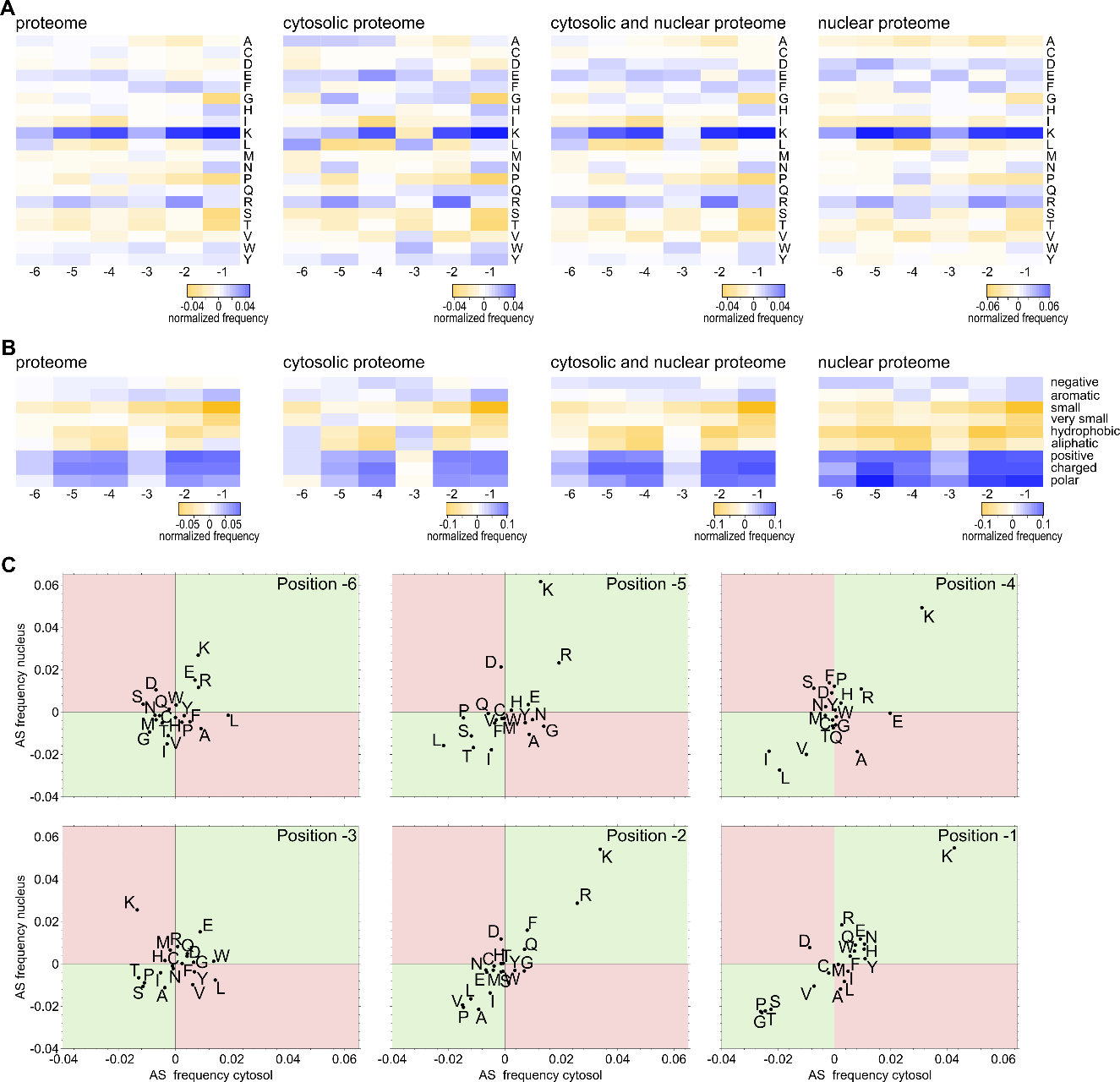


**Supplementary Figure S6: Normalized frequency of C-terminal amino acids at the last six C-terminal positions within the proteome, cytosolic proteins, cytosolic and nuclear proteins together, and nuclear proteins.** The amino acid abundance was normalized to their abundance in the proteome to indicate higher or lower occurrence at C-termini. Values below zero (shades of orange) indicate under-representation at a position and values greater than zero (shades of blue) indicate over-representation. A) Amino acid frequency at C-terminal positions. B) Amino acid properties at C-terminal positions. C) Correlation graph of amino acid (AS) frequencies at the last six positions of cytosolic proteins versus nuclear proteins.


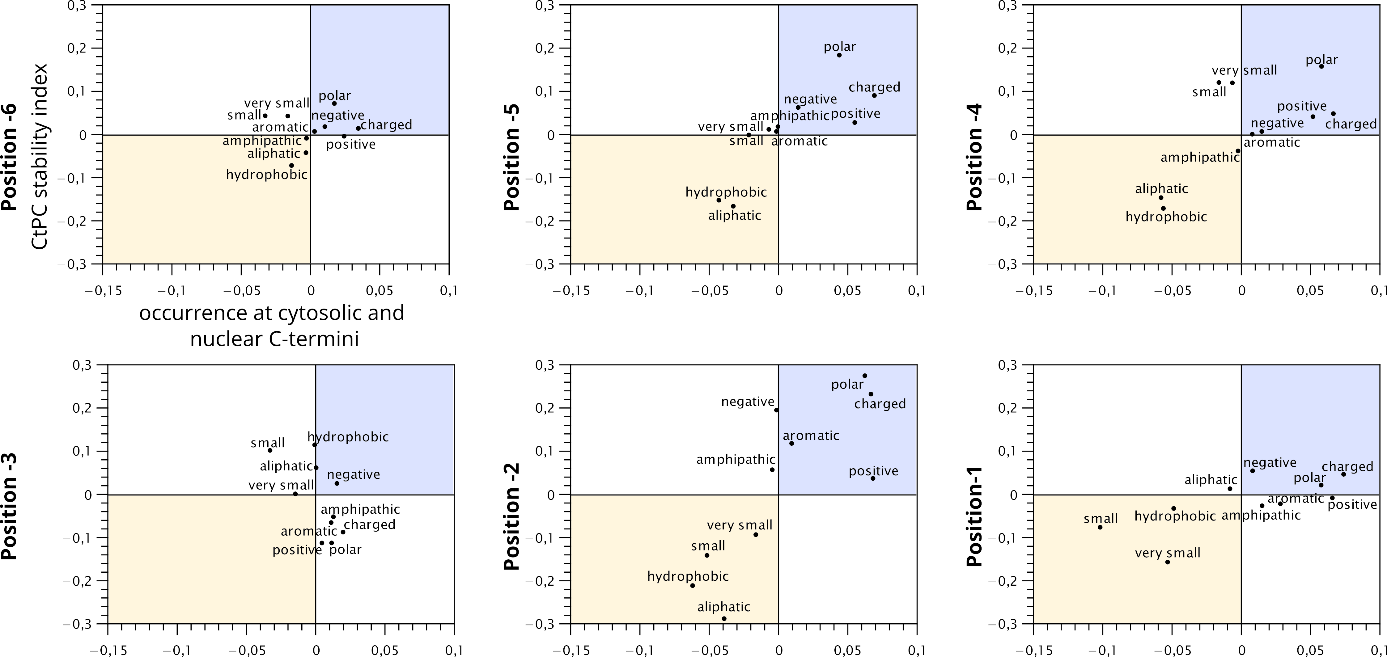


**Supplementary Figure S7: Correlation of the CtPC stability index with amino acid properties in cytosolic and nuclear proteins.** The normalized distribution of amino acid categories (grouped in accordance with their chemical properties) at the specific positions in the cytosol and nucleus were correlated to the distribution in CtPC-degrons.


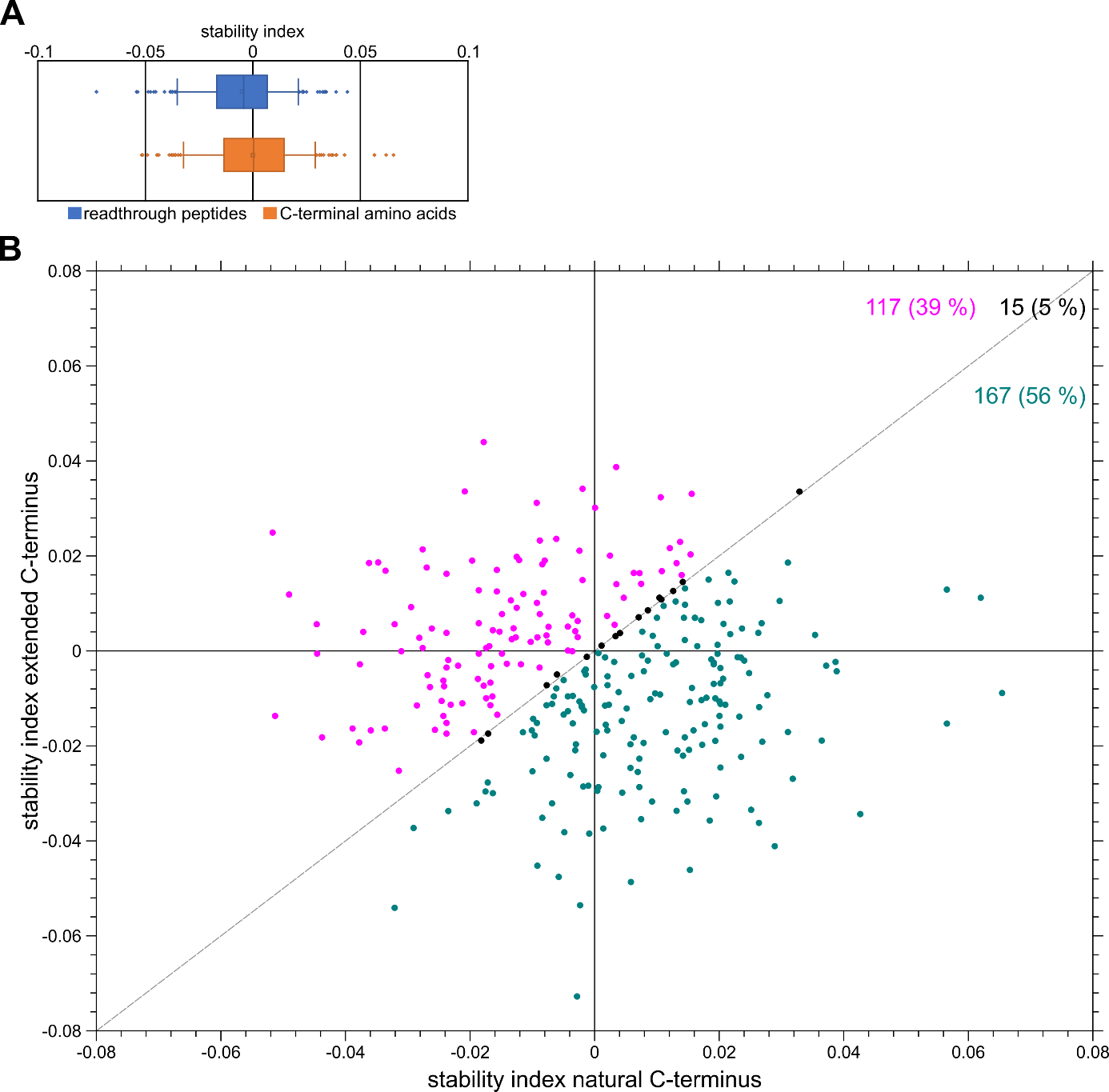


**Supplementary Figure S8: Stability indices of the last six amino acids of first frame read-through extensions in 299 highly produced proteins compared to the C-termini of the endogenous proteins.** The list of proteins was obtained from Kleppe et al (1). (A) Comparison of the stability index of extended proteins with endogenous protein ends. The last six amino acids of each variant were used for the calculation. The mean value is indicated by an open square, the median by a line within the boxplot. The box comprises values between 25 and 75 %, the whiskers from 5 % to 95 %. Outliers are indicated by a diamond. (B) Correlation of natural C-termini stability indices with C-terminal protein extensions due to translational stop-codon read-through. A pink color indicates proteins with an increased stability index after read-through extension, black color indicates proteins without change, and dark cyan color indicates proteins with decreased stability index after read-trough extension.


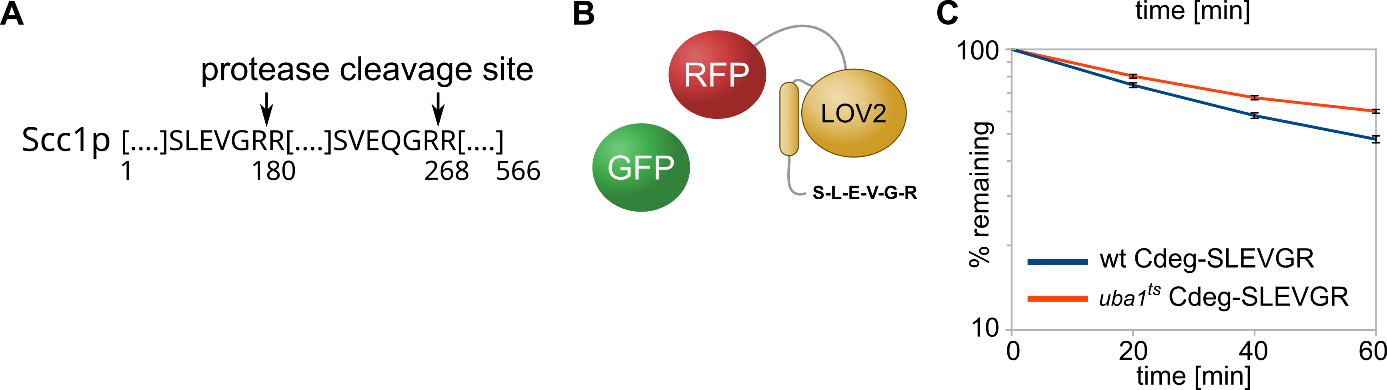


**Supplementary Figure S9: Characterization of a potential C-degron in yeast.** (A) Illustration of the protease Esp1 cleavage sites within the protein Scc1p. (B) Fluorescence-based system to investigate a destabilizing influence of the last six amino acids of the N-terminal fragment of the protein Scc1p after Esp1 cleavage (fusion construct: RFP-LOV2-SLEVGR). (C) Cycloheximide chase experiment of RFP-LOV2-Cdeg^SLEVGR^ in *uba1*^ts^ strain under blue light. Cells were incubated for 3 h at 25 °C followed by 2 h at restrictive temperature (37 °C) in darkness. Protein translation was inhibited using cycloheximide and cells were then incubated for 1 h in blue light while samples were collected. Error bars: SEM. The error bars indicate the standard deviation.


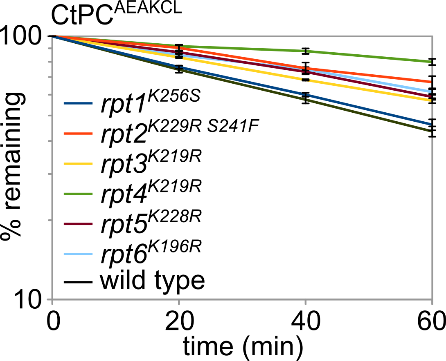


**Supplementary Figure S10***:* **Characterization of CtPC-degron degradation.** Cycloheximide chase analysis of CtPC-AEAKCL in Sub62 (wild type) and *RPT1-6* mutant strains. The chases were performed as described in Figure 3. The error bars indicate the SEM.


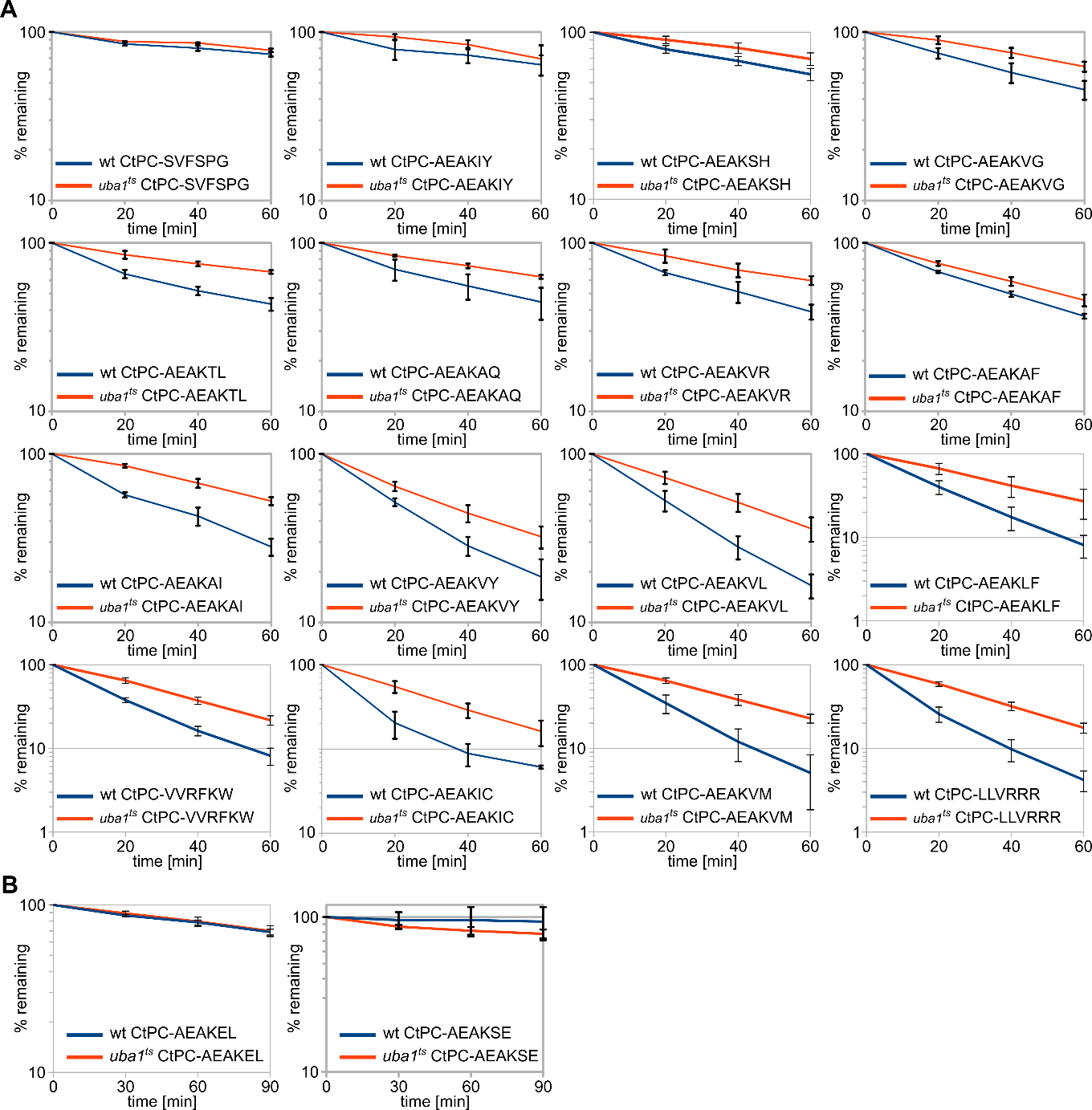


**Supplementary Figure S11: Degradation of CtPC-degrons requires Uba1.** Graphs show the cycloheximide chase analysis of selected CtPC-degrons in JD47-1 (wild type) and JD77 (*uba1*^ts^) strain. The analysis was performed as described in Figure 3. The error bars indicate the SEM. (A) Analysis of destabilized CtPC variants (as indicated). (B) Analysis of the stable variants CtPC^AEAKEL^ and CtPC^AEAKSE^.


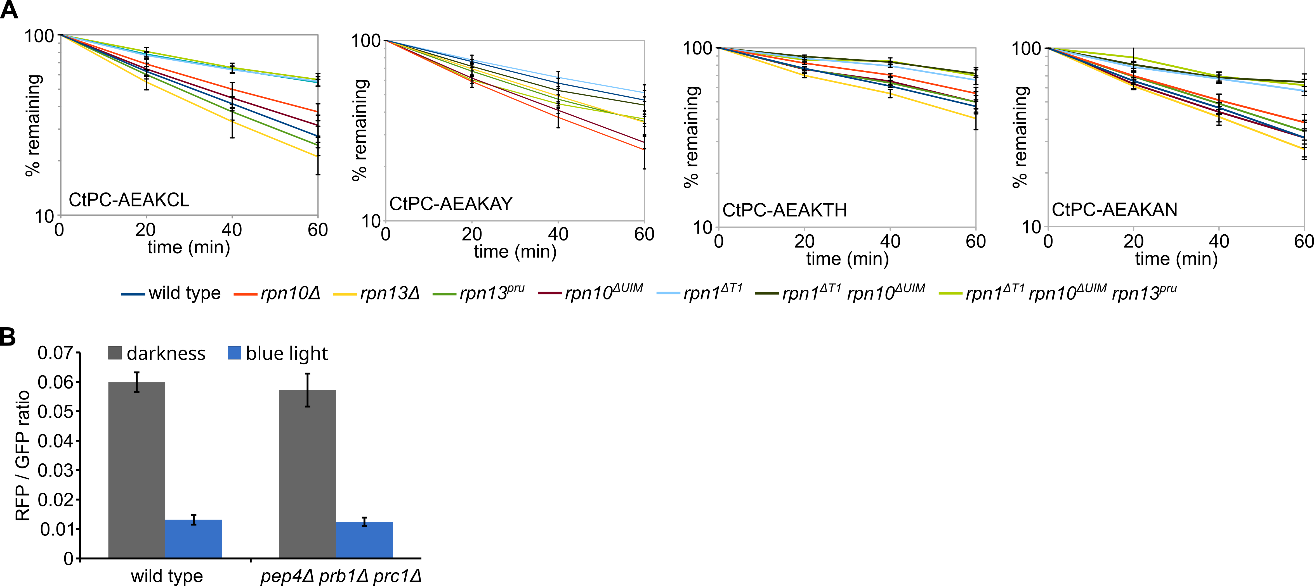


**Supplementary Figure S12: Degradation of CtPC-degrons requires proteasomal ubiquitin receptors.** A) Cycloheximide chase analysis in YYS40 (wild type) and ubiquitin receptor deletion strains YCMH8 (*rpn13^Δpru^*), YNDN1 (*rpn10Δ*), YNDN2 (*rpn13Δ*) and strains expressing mutated variants of ubiquitin receptors: YDAK34 (*rpn10^ΔUIM^*), YDAK36 (*rpn1^ΔT1^*), YSH35 (*rpn1^ΔT1^ rpn10^ΔUIM^*), YDAK47 (*rpn13^Δpru^ rpn10^ΔUIM^ rpn1^ΔT1^*)*.* The chases were performed as described in Figure 3. The error bars indicate the SEM. B) Analysis of CtPC^AEAKAY^ degradation in a yeast strain with inactivated vacuolar proteases. The graph shows fluorescence analysis by flow cytometer of CtPC^AEAKAY^ in W303-1B (wild type) and CBO18 (*pep4Δ prb1 Δ prc1 Δ*) strain. The analysis was performed as described in Figure 1. Error bars: standard deviation (n=8).


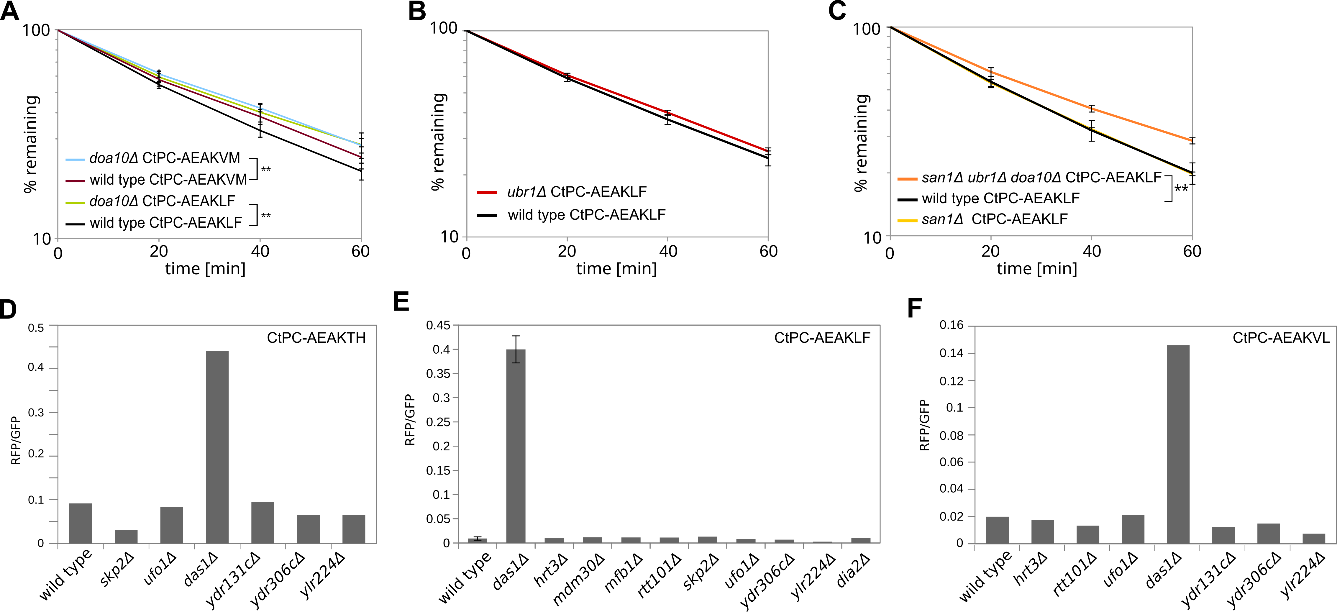


**Supplementary Figure S13***:* **Identification of E3s involved in CtPC degradation and test of substrate recognition subunits (SRS).** (A) Cycloheximide chase of CtPC-AEAKVM and CtPC-AEAKLF within MHY1631 (*doa10Δ*) strain. Significance was determined by Wilcoxon Signed-Rank Tests for Paired Samples. Cycloheximide chase of CtPC-AEAKLF in (B) ESM356 (wild type) and YCT1084 (*ubr1Δ*) and (C) in BY4741 (wild type), YHUM3335 (*san1Δ*) and YSH31 (*san1Δ doa10Δ ubr1Δ*) strains. The error bars indicate the SEM. (D-F) RFP/GFP ratios of CtPC-degrons after 5 h incubation in blue light in different SRS-deletion strains. Error bars indicate the standard deviation. The error bars indicate the SEM.

1* *[ ]* *10

1 p7 * *-QIFRVI---* *

2 p11 * *-ELYKQI---* *

3 p20 * *--LYKQIN--* *

4 p16 * *KQIYHS----* *

5 p19 * *FDIRKT----* *

6 p10 * *----RAYRCS* *

7 p3 * *---CKRLTC-* *

8 p2 * *-GTKKMI---* *

9 p14 * *----KAAGVG* *

10 p21 * *-AEWKEL---* *

11 p1 * *-AEAKIC---* *

12 p6 * *-AEAKAN---* *

13 p9 * *-AEAKAI---* *

14 p15 * *-AEAKAQ---* *

15 p22 * *-AEAKTS---* *

16 p5 * *-AEAKCL---* *

17 p13 * *-AEAKTL---* *

18 p24 * *-AEAKVL---* *

19 p25 * *-AEAKTH---* *

20 p18 * *-AEAKVG---* *

21 p17 * *-AEAKSI---* *

22 p12 * *-AEAKSH---* *

23 p4 * *-AEAKVM---* *

24 p8 * *-AEAKSM---* *

25 p23 * *-AEAKLF---* *

consensus/100% * *....+.....* *

consensus/90% * *...h+.....* *

consensus/80% * *.t.hK.h...* *

consensus/70% * *.s.tKsh...* *

hydrophobic => h { A, C, F, G, H, I, K, L, M, R, T, V, W, Y }

positive => + { H, K, R }

small => s { A, C, D, G, N, P, S, T, V }

turnlike => t { A, C, D, E, G, H, K, N, Q, R, S, T }

**Supplementary Figure S14: Consensus sequence of C-terminal CtPC degrons requiring Das1 for efficient degradation.** The sequence alignment was done with ClustalX and the consensus sequence was obtained with MViev (2, 3).

**Literature**

1. Kleppe, A. S., and Bornberg-Bauer, E. (2018) Robustness by intrinsically disordered C-termini and translational readthrough. *Nucleic Acids Res.* **46**, 10184–10194

2. Brown, N. P., Leroy, C., and Sander, C. (1998) MView: a web-compatible database search or multiple alignment viewer. *Bioinformatics*. **14**, 380–381

3. Madeira, F., Pearce, M., Tivey, A. R. N., Basutkar, P., Lee, J., Edbali, O., Madhusoodanan, N., Kolesnikov, A., and Lopez, R. (2022) Search and sequence analysis tools services from EMBL-EBI in 2022. *Nucleic Acids Res.* **50**, W276–W279
